# Design of a Mechatronics Model of Urinary Bladder and Realization and Evaluation of Its Prototype

**DOI:** 10.1155/2019/9431781

**Published:** 2019-12-14

**Authors:** Zhao Zhuo, He Dan, Li Gensong, Shi Ping, Pang Xining

**Affiliations:** ^1^Department of Stem Cells and Regenerative Medicine, Key Laboratory of Cell Biology, Chinese Ministry of Public Health and Key Laboratory of Medical Cell Biology, Chinese Ministry of Education, China Medical University, Shenyang, 110122 Liaoning, China; ^2^Department of Experimental Center of Functional Disciplines, China Medical University, Shenyang, 110122 Liaoning, China; ^3^Department of Academician Expert Workstation and Liaoning Province Human Amniotic Membrane Dressings Stem Cells and Regenerative Medicine Engineering Research Center, Shenyang Amnion Biological Engineering Technology Research and Development Center Co., LTD, Shenyang, 110000 Liaoning, China

## Abstract

Annually, there are many bladder cancer patients undergoing radical cystectomy (RC) with urinary diversion worldwide. Until 2019, intestinal cystoplasty is still the gold standard for bladder replacement, but this therapy is always associated with severe complications. An ideal bladder substitute without using intestinal tissue remains a challenge today. In this work, an artificial mechatronics bladder (AMB) as a brand new bladder replacement approach is developed. We studied the main physiological function characteristics of a natural urinary bladder from teaching books and relevant papers. According to these characteristics, we completed an overall design of AMB and made a prototype in lab. The prototype successfully realized the functions of a natural bladder in vitro. It can expand to store urine in real time when urine is flowing into it. It can send a urination alarm when it is fully filled and can void urine automatically after receiving remote control signals. According to relevant papers and our test experience, if the prototype could be smaller and lighter and manufactured with good biocompatibility materials such as PTFE, we think it is possible for AMB to be implanted in an animal's body, and we deduce AMB could realize the functions of a natural urinary bladder in vivo. After thorough validation from animal testing, we hope AMB can be a good clinical option for bladder removal patients in the future.

## 1. Introduction

Bladder cancer is the most common malignancy of the urinary tract [1]. Annually, over 7000 patients are estimated to undergo radical cystectomy (RC) with urinary diversion in the United States [2]. However, patients have to face a multitude of complications [3] and live a suboptimal quality of life after RC, because this traumatic event is often associated with significant changes in body image, urinary and sexual functions, interpersonal relationships, and psychosocial stress outcomes [4].

For almost a century, genitourinary science has been pursuing an ideal bladder substitute which has traditionally been done with intestinal segments. “Is it possible that we are no longer confined to the bowel for bladder replacement [5]?” However, until 2019, intestinal cystoplasty remains the gold standard [5–7]. Bladder replacement or expansion without using intestinal tissue is still a challenge, despite progress in the manufacture of biomaterials and the development of cell therapy [6].

To develop a new solution different from intestinal cystoplasty, we attempt to make an artificial mechatronics bladder (AMB) as a new ideal bladder substitute. In the process of searching literature, we found Rohrmann et al. [8] presented a spherical silicon rubber bladder (SRB) in 1996 which reports that the SRB can efficiently collect urine by creating a negative pressure and discharge urine by manually compressing the belly of the sheep. It was implanted in 5 sheep and performed well in vivo for a minimum of 147 days and a maximum of 595 days. From Rohrmann's report, we got three pieces of important information:

1. A negative pressure could guarantee urine to fill the bladder
2. Urine could be discharged by manually compressing the belly to contract the bladder
3. Reflux problem must be considered in the design of artificial bladder

According to Rohrmann's report, we believe it is possible to make an AMB, what we need to do is to make SRB be able to monitor the volume of urine by using appropriate sensors and discharge automatically by using mechanical structure rather than manually compressing.

So far, we have completed the overall design of AMB and made a prototype in lab. The prototype has already realized the functions of a natural bladder in vitro. We hope AMB can realize the functions of a natural bladder in vivo, and in the future, it can be a good clinical option for bladder removal patients.

## 2. Overall Design of AMB

From teaching books and relevant papers [9, 10], we got the information that the main physiological functions of a natural urinary bladder are to store and void urine. The bladder relaxes while urine is flowing into it; this can help urine flow into the bladder and at the same time keeps the bladder internal pressure stable. When the bladder is full, it will send urination signal to the brain and void urine by contraction of the urinary bladder smooth muscle. In addition, during urination, the ureter closes to prevent the reflux of urine and the urethral sphincter opens to allow the urine to get out.

According to the characteristics of a natural urinary bladder above, we can design our AMB as below:

1. There needs to be a urinary reservoir which can expand to store urine and contract to void urine
2. The urinary reservoir is required to expand automatically while urine is flowing into it
3. When the urinary reservoir is full, AMB needs to send out a urination alarm and patients can control AMB to void urine
4. AMB needs valves to prevent the reflux of urine and uroclepsia like ureter and urethral sphincter

**Figure 1:**
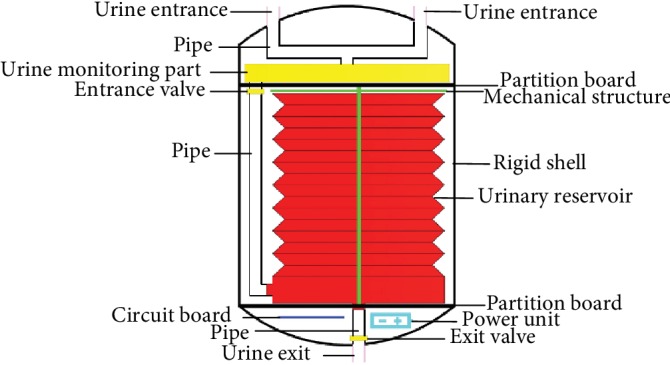
Overall schematic diagram of AMB.

So according to the above, we designed AMB as Figure 1 shows; it consists of five parts which are an outerwear part, a urinary reservoir, an expandable and contractable mechanical structure, a control system, and a power unit.

### 2.1. Outerwear Part

The outerwear part should include two urine entrances, one urine exit, a rigid shell, two partition boards, and several pipes. The urine entrances are connected to both ureters, and the urine exit is connected to the urethra. The rigid shell is used to protect its internal subassemblies from the influence of the inner environment and keep them working stably.

From relevant papers [9, 10], we got the information that the urinary bladder is able to store urine up to 500 ml in the normal adult. Theoretically, the full natural bladder could be considered as a sphere, and the diameter of a 500 ml sphere is about 100 mm, so we design the height and diameter of the rigid shell both fewer than 90 mm so that AMB could be small enough to be implanted in the pelvic cavity. The partition boards divide the inside of the shell into different workspaces in order to place different subassemblies of AMB. The pipes, as a path for urine to transport, connect different parts of AMB together inside the shell. According to relevant papers [8, 11–15], we prefer extended polytetrafluoroethylene (ePTFE) as an ideal material to make the urine entrances and exit and polytetrafluoroethylene (PTFE) to make the rigid shell.

### 2.2. Urinary Reservoir

**Figure 2:**
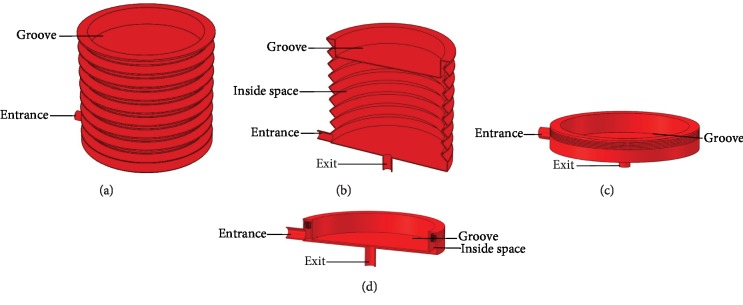
Urinary reservoir. (a) A fulfilled urinary reservoir. (b) Sectional view of the fulfilled urinary reservoir. (c) An emptied urinary reservoir. (d) Sectional view of the emptied urinary reservoir.

As shown in Figure 2, the urinary reservoir is an expandable and contractable cylinder which maximum height is 70 mm and diameter is 80 mm. On the top of the urinary reservoir, there is a groove. The height of the groove is 10 mm, and its diameter is 70 mm.

From teaching book and relevant papers [16, 17], we got the information that commonly, the voided urine volume of a normal person is about 300 ml. According to this information and the size of the rigid shell, we design the capacity of the urinary reservoir to 300 ml with 70 mm height, 80 mm diameter, and 10 mm groove. Under common conditions, this design could meet the requirement of normal urine volume; meanwhile, it is small enough to be implanted in the pelvic cavity with the outerwear part and mechanical structure. When the urinary reservoir is emptied as Figure 2 shows, the groove will fill the inside space of the urinary reservoir, and the surplus inside space is an annulus cylinder which volume is theoretically less than 12 ml. So the urinary reservoir has very little residual urine when it is emptied. The urinary reservoir is the core mechanical part of our AMB; the mechanical structure and outerwear part are both designed based on its shape and size.

### 2.3. Mechanical Structure

The design of the mechanical structure is open-minded; it should meet the conditions below:

1. The mechanical structure is able to realize the function of expanding and contracting the urinary reservoir
2. The mechanical structure should be small enough to be put inside the rigid shell with other relevant subassemblies

Any available mechanical structure that can meet these conditions is OK. In our opinion, the simpler structure, the better. A simpler structure usually has smaller size and lighter weight; small size and light weight are essential conditions for implantation. Meanwhile, it should meet the requirement that the mechanical structure could realize its function steadily for long-term or permanent use. So to sum up, a better design of the mechanical structure is a design which simultaneously meets the three requirements of smaller, lighter, and available for long-term or permanent use.

Our current mechanical structure is able to realize the function of expanding and contracting the urinary reservoir, and it could be fixed inside the rigid shell. Our design is shown in Section 3; we use a stepping motor to drive the mechanical structure.

### 2.4. Control System

The control system is used to control the whole AMB working process which starts with the flowing of urine into AMB and ends with discharging urine out of the body. The control system includes a urine monitoring part, two electromagnetic valves, and a circuit board.

The urine monitoring part is used to monitor when the urine is flowing into the urinary reservoir and whether the urinary reservoir is filled. The urine monitoring part includes a small soft balloon of 1-2 ml capacity, a liquid flow meter, and a photoelectric switch. To guarantee the urine monitoring part to realize its function, the small balloon must be soft enough to be passively filled by the pressure of the kidney and the ureter. When urine flows into the balloon, the liquid flow meter will sense the flow and send pulses to the circuit board. Once the urine fills the balloon, the circuit board will then automatically control the entrance valve to close and the stepping motor to rotate. The rotating stepping motor will drive the mechanical structure to take the urinary reservoir to expand; this will create a negative pressure to assist the urine to flow into the urinary reservoir from the small balloon. When the balloon barely has urine inside, the stepping motor stops rotating and the entrance valve opens again. This process repeats many times until urine is about to fill the urinary reservoir. When the urinary reservoir is about to be filled, it triggers the photoelectric switch to send a signal to the circuit board. The circuit board then will send an alarm signal to patients, and patients need to send back a urination signal to the circuit board. After receiving the urination signal, the circuit board will control the stepping motor to rotate reversely, the entrance valve closes to prevent reflux, and the exit valve opens like urethral sphincter. This time, the stepping motor drives the mechanical structure to take the urinary reservoir to contract to void urine. When the urinary reservoir is empty, the exit valve closes and the stepping motor stops rotating.

In our design, discharging anytime is also considered. Whether or not patients receive an alarm signal, they can send a signal to control AMB to discharge whenever they want. The program algorithm is as below.

The original state of the system is empty. As urine enters into the reservoir, the circuit board counts the rotating time of the stepping motor. Each time the stepping motor rotates for 1 second. When the circuit board receives the discharge anytime signal, it would control the stepping motor to rotate reversely for the duration of the count time. For example, if the rotating time of entering is 20 which means the stepping motor rotates 20 times, meanwhile, the circuit board receives the discharge anytime signal, it would control the stepping motor to rotate reversely for 20 seconds; the rotating of the stepping motor would take the mechanical structure back to its original state which stands for empty.

**Figure 3:**
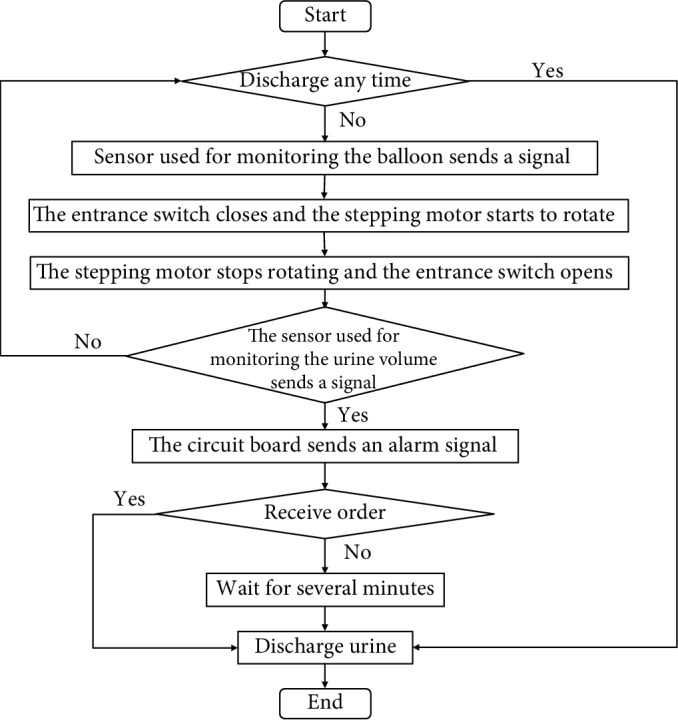
The work flow diagram of the system.

Figure 3 is the work flow diagram of the system.

### 2.5. Power Unit

In our design, the power unit consists of a battery and a wireless charging system. It is desired that the battery can provide power to AMB for at least 48 hours and the wireless charging system can charge the battery anywhere anytime. So far, in lab, we have achieved to charge a battery wirelessly by two antennas. However, the relevant paper [18] indicates this wireless energy transfer could cause heating in tissue because electromagnetically, tissue is a lossy dielectric material with properties described by permittivity and conductivity; wireless energy transfer technology seems still far away from clinical application. So under current technology condition, it still seems a better choice to put the battery outside the body.

## 3. Realization of the Prototype

In lab, we have made a prototype of AMB which consists of two parts. One part is to realize the functions of natural urinary bladder, we call it main body. The other part is to control and provide power, we call it control system.

**Figure 4:**
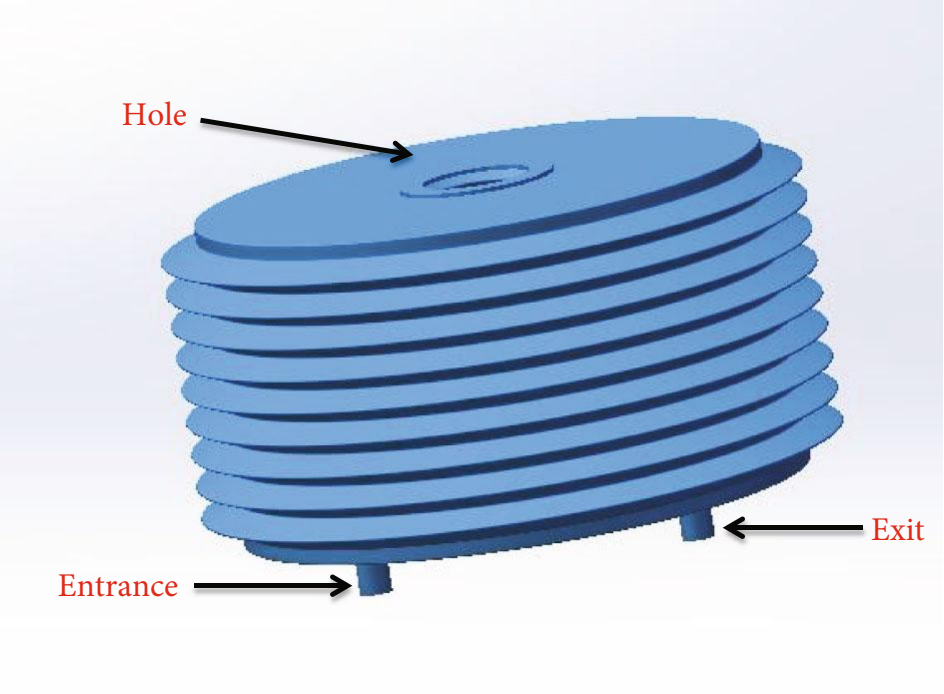
The urinary reservoir.

**Figure 5:**
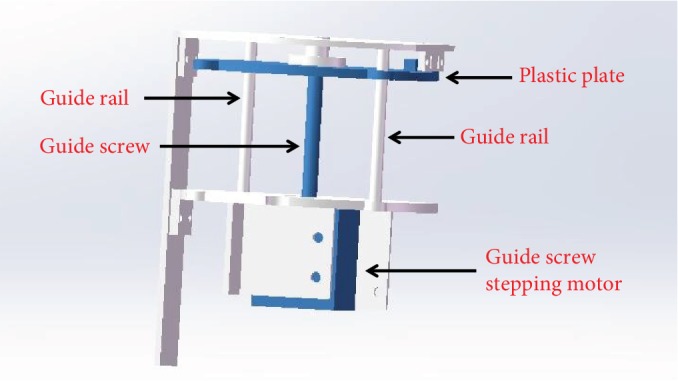
The mechanical structure.

Because of the fund limitation, the prototype cannot realize the overall design perfectly in lab. According to actual conditions, we designed the urinary reservoir and the mechanical structure as Figures 4 and 5 show. The height of the urinary reservoir is 40 mm, the diameter is 80 mm, and the cubage is less than 200 ml. The guide screw goes through the hole of the urinary reservoir. The plastic plate is fixed with the urinary reservoir. The rotating of the guide screw stepping motor can take the plastic plate to move up and down along the guide rail, so the plastic plate can take the urinary reservoir to expand and contract.

**Figure 6:**
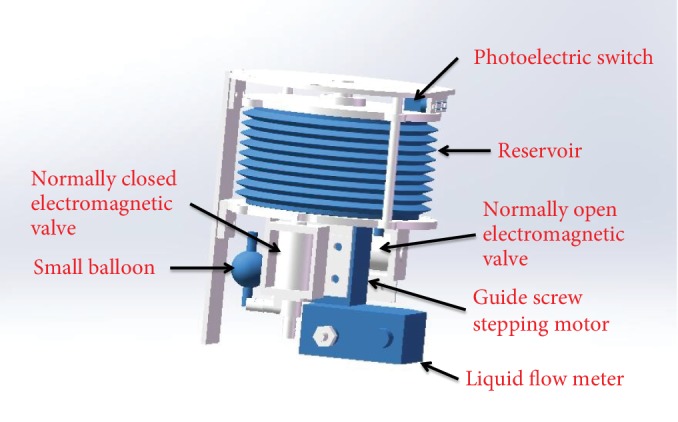
The inside of the main body.

**Figure 7:**
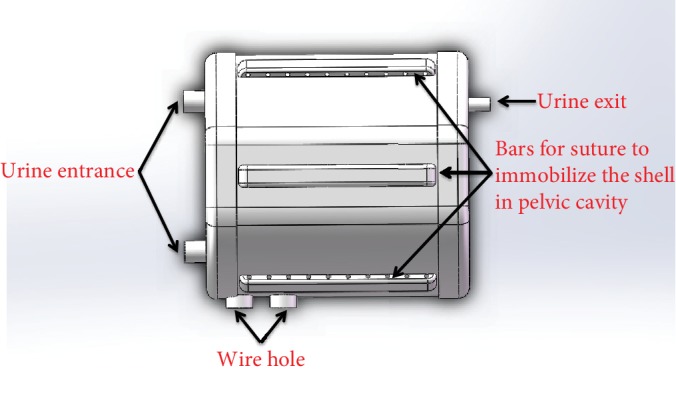
The shell of the main body.

We designed the main body as Figures 6 and 7 show. When the reservoir is fully expanded, the photoelectric switch is triggered to send a signal to the circuit board and the circuit board will make an alarm sound to tell patients that the reservoir is full. The shell of the main body has five bars that are used to immobilize AMB in the pelvic cavity. The diameter and height of the shell are both about 110 mm.

**Figure 8:**
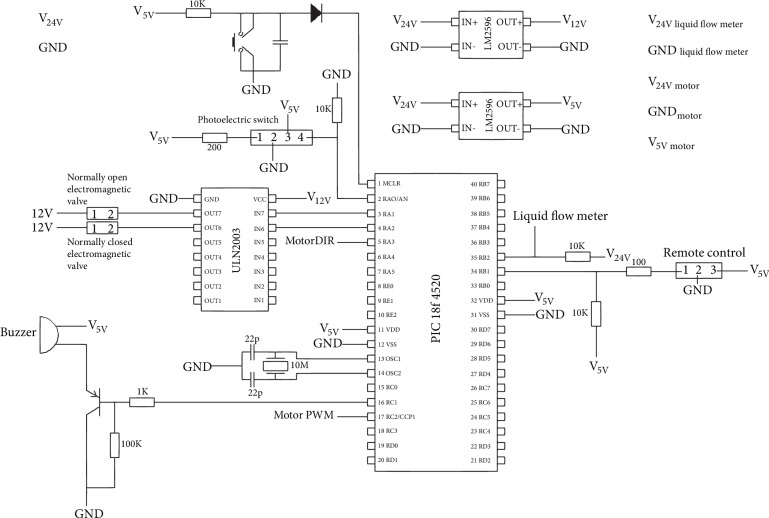
The design diagram of circuit board.

We designed the circuit board as Figure 8 shows. The microchip is PIC 18f4520. The microchip's pin RA1 is used to control the normally open electromagnetic valve. RA2 is used to control the normally closed electromagnetic valve. RC1 is used to control the buzzer. RC2 and RA3 are used to control the stepping motor, RC2 is used to output pulse, and RA3 is used to control the rotating direction. RB2 is used to receive the pulses of the liquid flow meter. RB1 is used to receive the remote signal. RA0 is used to receive the signal of the photoelectric switch. The voltage of the circuit board is 24 V. The program work flow of the microchip is as Figure 3 shows.

When urine is flowing into the small balloon, the liquid flow meter sends pulse signals to the microchip and the microchip counts the number of pulse signals. When the number equals to 200 (one pulse is generated when 5 *μ*l urine flows through the liquid flow meter), the normally open electromagnetic valve will close to prevent the urine continually flowing into the balloon and the stepping motor will rotate to make the reservoir expand a little, so the urine in the small balloon will flow into the reservoir because of the negative pressure created by the expanding of the reservoir. When the small balloon is empty, the normally open electromagnetic valve opens and the urine flows into the balloon again. This process repeats many times until the reservoir triggers the photoelectric switch to send a signal to the circuit board, and the buzzer on the circuit board will raise an alarm at this moment. While hearing the alarm, patients can send a signal with remote control to the circuit board and the microchip on the circuit board will control the normally open electromagnetic valve to close to prevent reflux, control the normally closed electromagnetic valve to open like urethral sphincter, and control the stepping motor to rotate inversely to make the reservoir contract until the reservoir is empty. When the reservoir is empty, the stepping motor stops rotating, the normally closed electromagnetic valve closes, and the normally open electromagnetic valve opens. Urine starts to flow through the liquid flow meter into the balloon again.

**Figure 9:**
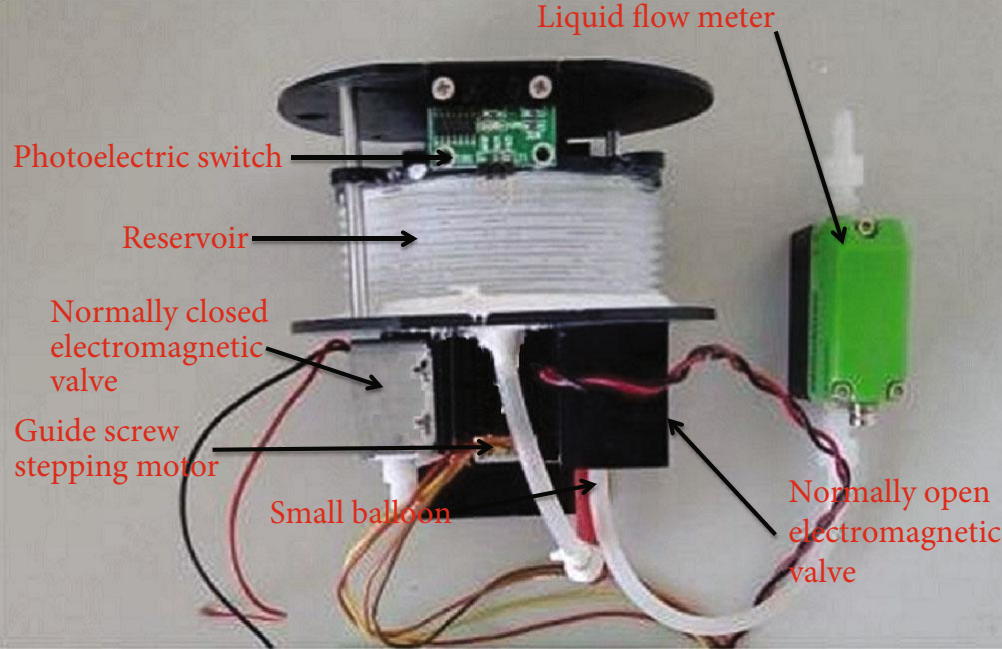
The inside of the prototype's main body.

**Figure 10:**
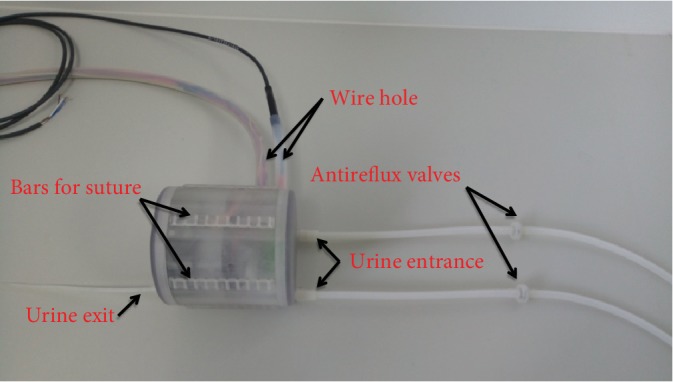
The main body of the prototype.

**Figure 11:**
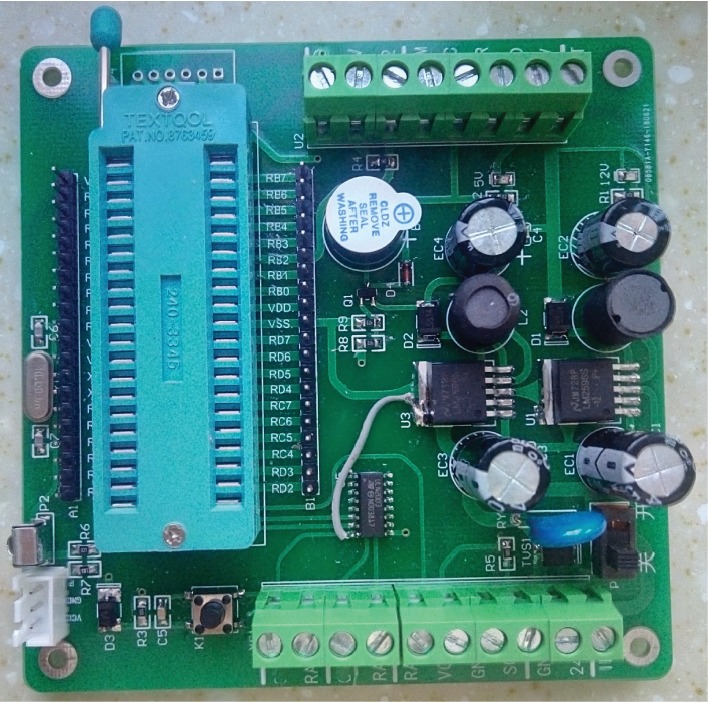
The circuit board of the prototype.

Figure 9 shows the inside of the prototype's main body we made, Figure 10 shows the main body of the prototype we made, and Figure 11 shows the circuit board we made.

**Figure 12:**
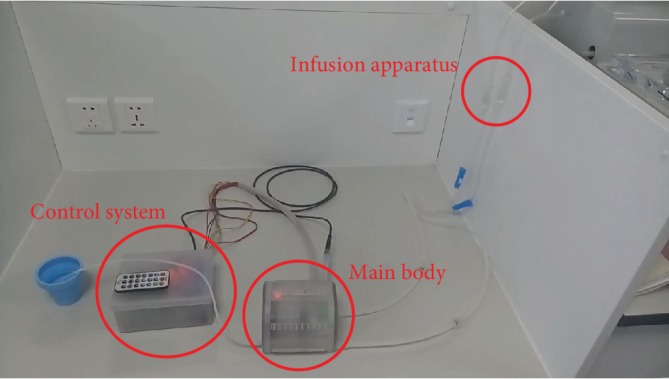
Test of the prototype.

Figure 12 shows the final test we did. For normal adults, median urine generation rate is about 1.42 l per day [19] which indicates that the average speed of urine that flows into the urinary bladder is about 1 ml/min, so we use infusion apparatus to simulate urine that flows into the urinary bladder. During urination, the internal pressure of a natural bladder could reach 150 cm H_2_O [20] which is considered to be strong contractility. For ease of calculation, we assume that the cross-sectional area of the urethra is 1 cm^2^ which is obviously larger than the actual area. The conversion of internal pressure indicates the contraction pressure of a natural bladder is normally less than 0.15 kgf. Because the shell is rigid, standard atmospheric pressure should be considered too. So finally, we calculated the urination pressure for AMB is less than 1.2 kgf. The thrust that the mechanical structure generates is more than 1.5 kgf, so theoretically, the prototype could realize the urination function even if it was in vivo.  Table 1 shows the test results of the prototype.

## 4. Discussion

Antireflux valves shown in Figure 10 are very important subassemblies for the AMB prototype. The first time we tested the main body, we found the two urine entrances impacted on each other. When the pressure of the two entrances is not balanced, reflux occurs in the entrance with low pressure. This is what we did not expect in our original design. So we tried to use antireflux valves inserted at the two entrances, and the reflux problem was successfully solved.

Intestinal cystoplasty is currently the gold standard for bladder replacement [5–7], but this therapy is always associated with severe complications such as stone formation, urine leakage, and chronic infections [7]. In addition, other primary research directions for bladder replacement like tissue engineering and biomaterials are also facing many challenges currently [7]. This research is aimed at providing a mechatronics approach as a brand new strategy for bladder replacement with the intent to solve or avoid the problems that current approaches and strategies cause. Among all the papers we searched about artificial bladder, we have not found any similar research papers or reports about mechatronics bladder, so we think our team is the first one that attempts to develop AMB for bladder replacement.

We think there are two innovation points in our design. First, we used a cylindrical urinary reservoir but not a traditional spherical reservoir to store urine; the advantage of this design is the cylindrical reservoir is easy to be taken to expand and contract by simple mechanical structures, but a spherical reservoir is not that easy. Second, we find no papers or works have reported any method about how to monitor urine that flows into a natural bladder, so we invent a method that uses a small balloon and a liquid flow meter to solve this problem. When the small balloon is full, the cylindrical reservoir will expand to create a negative pressure to make the urine flow into the reservoir from the small balloon. When the balloon is empty, the reservoir will stop expanding, and urine flows into the balloon from ureters again. In this design, the manufacturing material of the reservoir must be harder than that of the balloon, or else the expanding reservoir would collapse so that it cannot create a negative pressure to make the balloon empty. In addition, in this design, we think a liquid flow meter is very suitable as a sensor because it is not affected by body posture while it is monitoring the urine flow.

Our test result reveals the prototype successfully realized the functions of a natural urinary bladder in vitro. It can automatically monitor urine flow and expand the reservoir to store urine, it can send out urination alarm when the reservoir is full, and it can automatically compress the reservoir to void urine after receiving the remote control signal. But due to the limitation of actual conditions, there remains a big gap between the prototype and the overall design. The main problem is that the stepping motor, the valves, and the liquid flow meter are a little big and heavy for AMB. If we had smaller and lighter subassemblies, the whole volume of the prototype could be smaller; at the same time, the cubage of the urinary reservoir could be bigger. This would be much closer to the overall design.

We think the overall design of AMB is a good model that can realize the functions of a natural urinary bladder. The key elements of the model are an expandable and contractable cylindrical reservoir and a monitoring part which is composed of a small balloon and a liquid flow meter. Valves that are used to prevent reflux and incontinence, and relevant mechanical structures and control system are also significant components. According to relevant papers [8, 21] and our test experience, if the prototype could be smaller and lighter and manufactured with good biocompatibility materials such as PTFE, we think it is possible for AMB to be implanted in an animal's body, and we deduce AMB could realize the functions of a natural urinary bladder in vivo. If AMB could realize the functions of a natural urinary bladder in vivo, we could even think about long-term or permanent clinical implantation.

## 5. Conclusion

In this paper, we have completed the overall design of AMB and made a prototype in lab. The prototype has already successfully realized the functions of a natural urinary bladder in vitro. It can expand to store urine in real time when urine is flowing into it. It can send a urination alarm when it is fully filled and can void urine automatically after receiving remote control signals. Based on this model, if we could improve the prototype, we think it is possible for the updated AMB to be implanted in an animal's body and realize its functions in vivo. After thorough validation from animal testing, we hope AMB could be a good clinical option in the future for bladder removal patients.

## Figures and Tables

**Table 1:** Test results of the prototype.

Test round	Entering speed		Entering duration	Discharge speed
	Left entrance	Right entrance		
1	1.1 ml/min	1.1 ml/min	30 min	60 ml/min
2	1.2 ml/min	0.8 ml/min	32 min	60 ml/min
3	1 ml/min	0.9 ml/min	32 min	60 ml/min

## Data Availability

Readers can send e-mail to 420725538@qq.com for the test video of the prototype.

## Supplementary Materials

Supplementary Materials Among these 3 files, one is the product brochure, one is datasheet of the sensor, and the other is datasheet of the sensor cable. The datasheet of the sensor cable provides us the introduction of the sensor modes (in the manuscript, we use “Volume Counter” mode) and the connection diagram. The other two files are mainly about the introductions of the sensor such as specification charts and patented technology.
