# Supplementary material for: Design of a Mechatronics Model of Urinary Bladder and Realization and Evaluation of Its Prototype
Source: Appl Bionics Biomech. 2019 Dec 14;2019:9431781. doi: 10.1155/2019/9431781 (PMC6948342; doi:10.1155/2019/9431781)
Supplement: Supplementary Materials — Among these 3 files, one is the product brochure, one is datasheet of the sensor, and the other is datasheet of the sensor cable. The datasheet of the sensor cable provides us the introduction of the sensor modes (in the manuscript, we use “Volume Counter” mode) and the connection diagram. The other two files are mainly about the introductions of the sensor such as specification charts and patented technology. [file 9431781.f1.zip › Sensirion_Liquid_Flow_Meters_Product_Brochure.pdf]

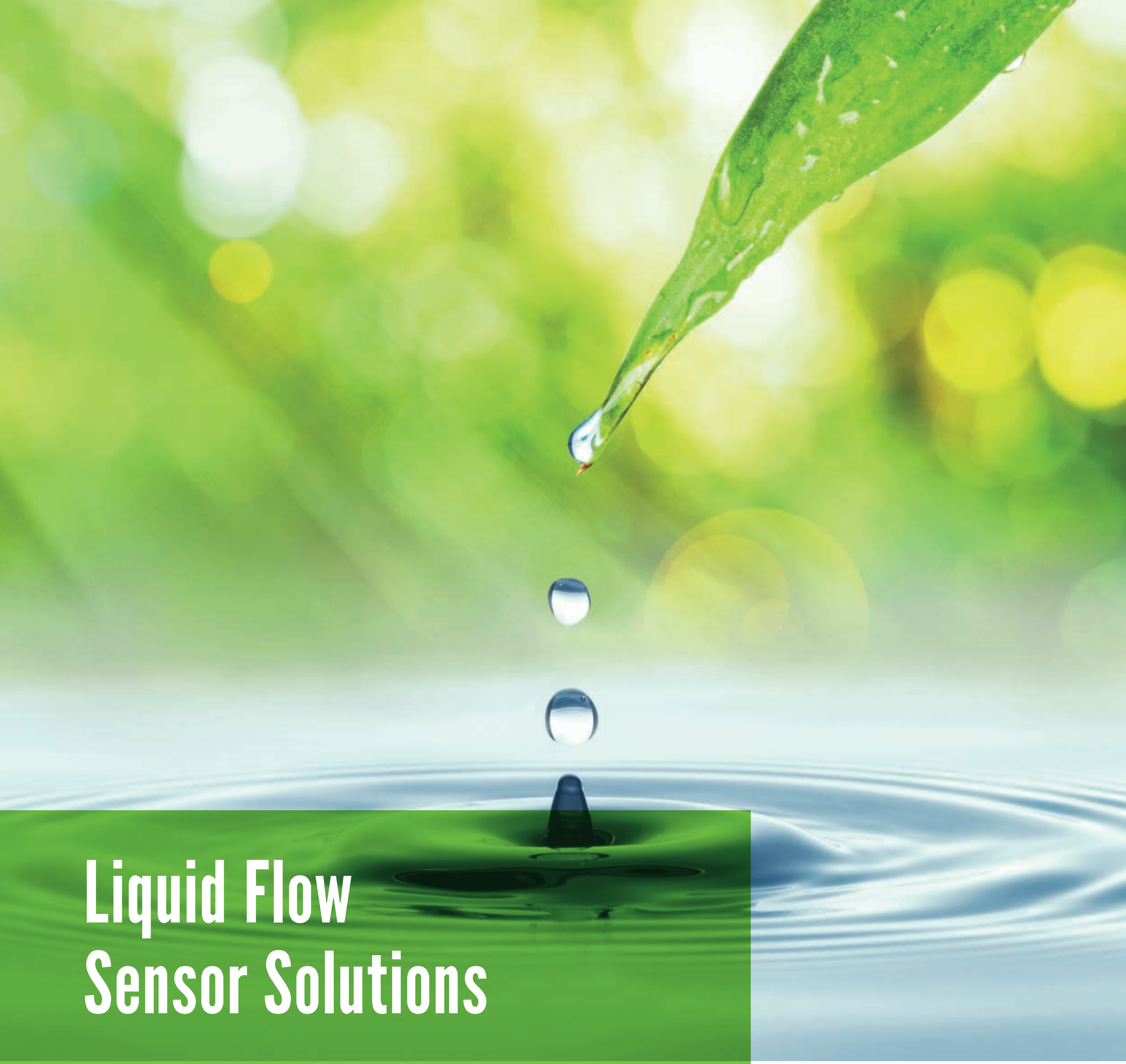

Liquid Flow Sensor Solutions

Experts for Smart Sensor Solutions

SENSIRION
THE SENSOR COMPANY

Inspiring Liquid Flow Sensor Technology

Sensirion's liquid flow meters establish new standards wherever monitoring of low liquid flow rates, liquid handling, and liquid dispensing is important. Our unique CMOSens® Technology allows measuring the liquid flow bidirectionally through the wall of the sensor's flow channel from hundreds of milliliters per minute down to single-digit nanoliters per minute. Applications in fields like medical devices, diagnostics as well as process and automation technology benefit daily from our safe and reliable sensor solutions.

For more information, please visit: www.sensirion.com/liquidflow

PATENTED TECHNOLOGY

Sensirion's CMOSens® flow sensor technology is based on a thermal microsensor and is most effective at very low flow rates. The key element in our products is an integrated digital CMOSens® microchip bonded to the outside of the sensor's flow channel, which measures precisely through the wall of the flow channel. Our liquid flow meters offer completely media-isolated flow sensing with no moving parts or obstacles in the flow path. We offer our customers the world's smallest and most precise liquid flow meters and inspire new designs and applications throughout all industries. High reliability and perfect media compatibility make our sensors ideal for use in medical and life sciences, diagnostics, factory automation and energy management applications.

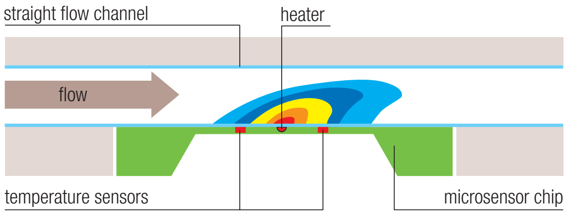

Flow measurement principle

FAST, SMALL, RELIABLE

Sensirion's standard liquid flow meters provide exceptional value for money and eliminate the need for investment in application-specific OEM sensor development. The inert wetted materials ensure excellent process compatibility, industry-standard fluidic fittings enable quick assembly into the fluidic line, and downmount fittings allow compact installation in manifold systems. Through the use of capillaries with different diameters, Sensirion's liquid flow meters cover flow rates over six orders of magnitude from single-digit nanoliters up to a few hundred milliliters per minute.

In addition to the sensor element, the CMOSens® chip integrates the complete digital intelligence and memory for signal linearization, temperature compensation and self-test algorithms. Different digital (I^2C, RS485, USB) or analog output options are available for easy testing and seamless integration. Please contact our experts to discuss possible options for customized solutions: info@sensirion.com

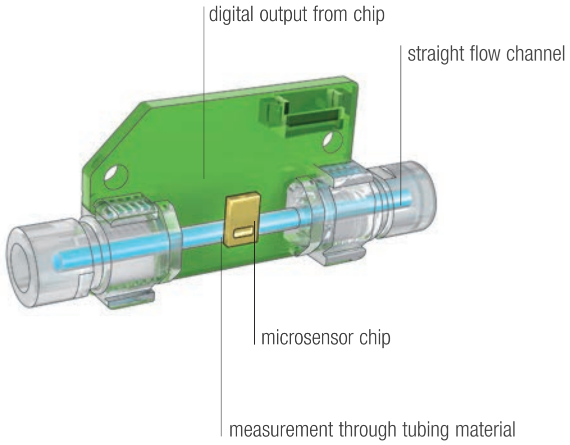

Schematic layout of a liquid flow meter

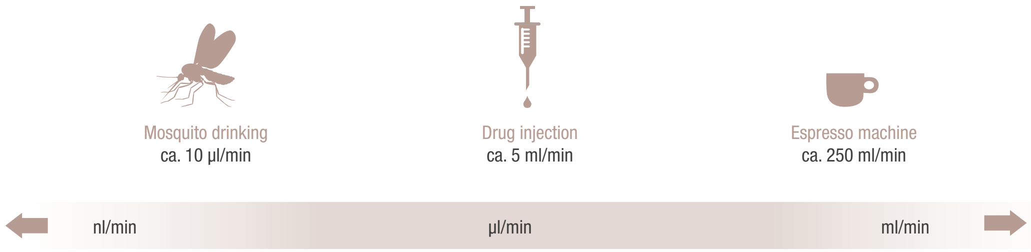

Sensirion is able to measure flow rates from single-digit nl/min up to several hundred ml/min.
Contact us if you require higher flow rates.

Selection of Sensirion's Liquid Flow Meters

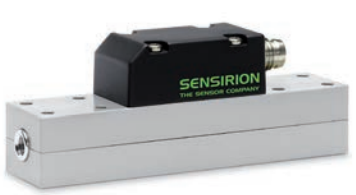

SLG SERIES

SLG liquid flow meters for ultra-low flow rates (down to single-digit nanoliters per minute) are the perfect solution for dynamic liquid flow monitoring at high pressures and/or low flow rates, such as cutting-edge UHPLC applications. The fused silica flow channel withstands pressures of up to 1200 bar.

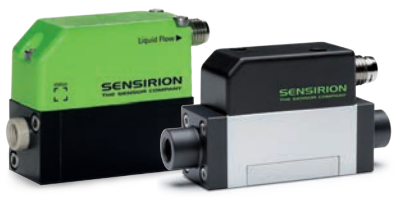

SLI / SLS SERIES

With Sensirion's flow meter technology in a protective housing, SLI / SLS liquid flow meters are ideal for laboratory work and for use in the automation industry. In combination with the SCC1 sensor cables, they provide RS485, analog or USB output for reliable communication in harsh environments.

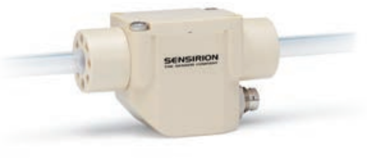

SLQ-QT500

A calibrated flow rate range of up to 120 ml/min and the exclusive use of high-purity wetted materials (quartz glass, PFA) make this sensor ideal for dispensing processes in demanding applications, such as the semiconductor industry, and for the measurement of liquids with high viscosity or containing particles.

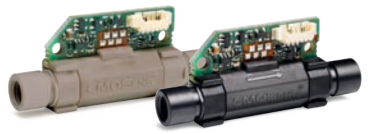

LG16 / LG01 SERIES

The small footprint of the LG16 series makes it the ideal choice for the integration of a liquid flow sensor into OEM devices. It is available in a digital version with I²C communication and a 0 - 5 V analog output version. The LG01 liquid flow switch is the perfect solution for the detection of flow, bubbles and leakages.

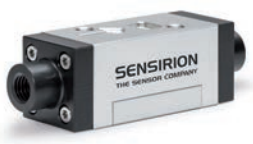

LS32-1500

The LS32-1500 comes in a compact housing with high mechanical robustness. The wetted materials provide exceptional chemical resistance. In addition to its suitability for the biomedical market, it also offers a cost-effective, compact and reliable solution for many applications with flow rates of up to 40 ml/min.

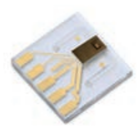

LPG10 SERIES

The LPG10 series represents our smallest liquid flow sensors and enables outstanding performance and speed in a very small form factor. The innovative design enables flow measurement in a planar microfluidic glass substrate. Downmount fluidic ports allow compact integration into manifold systems.

	Model	Full Scale Flow Rates	Accuracy of Measured Value	Bidirectional Operation	Output			Maximum Cable Length	Fluidic Connector Ports	Maximum Pressure
					Analog	RS485	I²C			
SLG	SLG-0025	1.5 µl/min H_2O	10 %		0 to 10V	✓	✓	> 100 m for RS485, 30 cm for I²C		1200 bar
	SLG-0075	5 µl/min H_2O	10 %	Yes	0 to 10V	✓	✓	> 100 m for RS485, 30 cm for I²C	Stainless steel 10-32 coned port for 1/16" OD tubing	1200 bar
	SLG-0150	8 µl/min H_2O	5 %		0 to 10V	✓	✓	> 100 m for RS485, 30 cm for I²C		500 bar
SLI	SLI-0430	80 µl//min H_2O<br>500 µl/min HC								50 bar
	SLI-1000	1 ml/min H_2O<br>10 ml/min HC	5 %	Yes	0 to 10V	✓	✓	> 100 m for RS485, 30 cm for I²C	¼-28 flat-bottom for 1/16" or 1/8" OD plastic tubing	12 bar
	SLI-2000	5 ml/min H_2O<br>80 ml/min HC								12 bar
SLS	SLS-1500	40 ml/min H_2O	10 %	Yes	0 to 10V	✓	✓	> 100 m for RS485, 30 cm for I²C	¼-28 flat-bottom for 1/8" OD tubing	12 bar
SLQ	SLQ-QT105	120 ml/min HC	10 %	Yes	0 to 10V	✓	✓	> 100 m for RS485, 30 cm for I²C	Super 300 type pillar fitting 4x3 mm	12 bar
	SLQ-QT500	120 ml/min H_2O<br>120 ml/min HC	10 %		0 to 10V	✓	✓	> 100 m for RS485, 30 cm for I²C	PFA tube with 6.35 mm (¼") OD, 4.35 mm ID	12 bar
LG16	LG16-0025	1.5 µl/min H_2O	10 %		0 to 5V		✓			200 bar
	LG16-0150	7 µl/min H_2O<br>70 µl/min HC			0 to 5V		✓		UNF 6-40 con for 1/32" OD tubing	200 bar
	LG16-0430	80 µl/min H_2O<br>500 µl/min HC		Yes	0 to 5V		✓	30 cm for I²C		100 bar
	LG16-1000	1 ml/min H_2O<br>10 ml/min HC	5 %		0 to 5V		✓			15 bar
	LG16-2000	5 ml/min H_2O			0 to 5V		✓		¼-28 flat-bottom for 1/16" or 1/8" OD plastic tubing	15 bar
	LG16-2000HC	80 ml/min HC					✓			15 bar
LS32	LS32-1500	40 ml/min H_2O	10 %	Yes			✓	30 cm for I²C	¼-28 flat-bottom for 1/8" OD tubing	12 bar
LPG10	LPG10-0500	100 µl/min H_2O	5 %	Yes			✓	30 cm for I²C	Downmount	10 bar
	LPG10-1000	1 ml/min H_2O					✓			3 bar
LG01	LG01-2000	Switch level: 0.25 ml/min or 4.5 ml/min	10 %	No	0V: no flow or bubble<br>5V: flow above switch level			3 m	¼-28 flat-bottom for 1/16" or 1/8" OD plastic tubing	3 bar

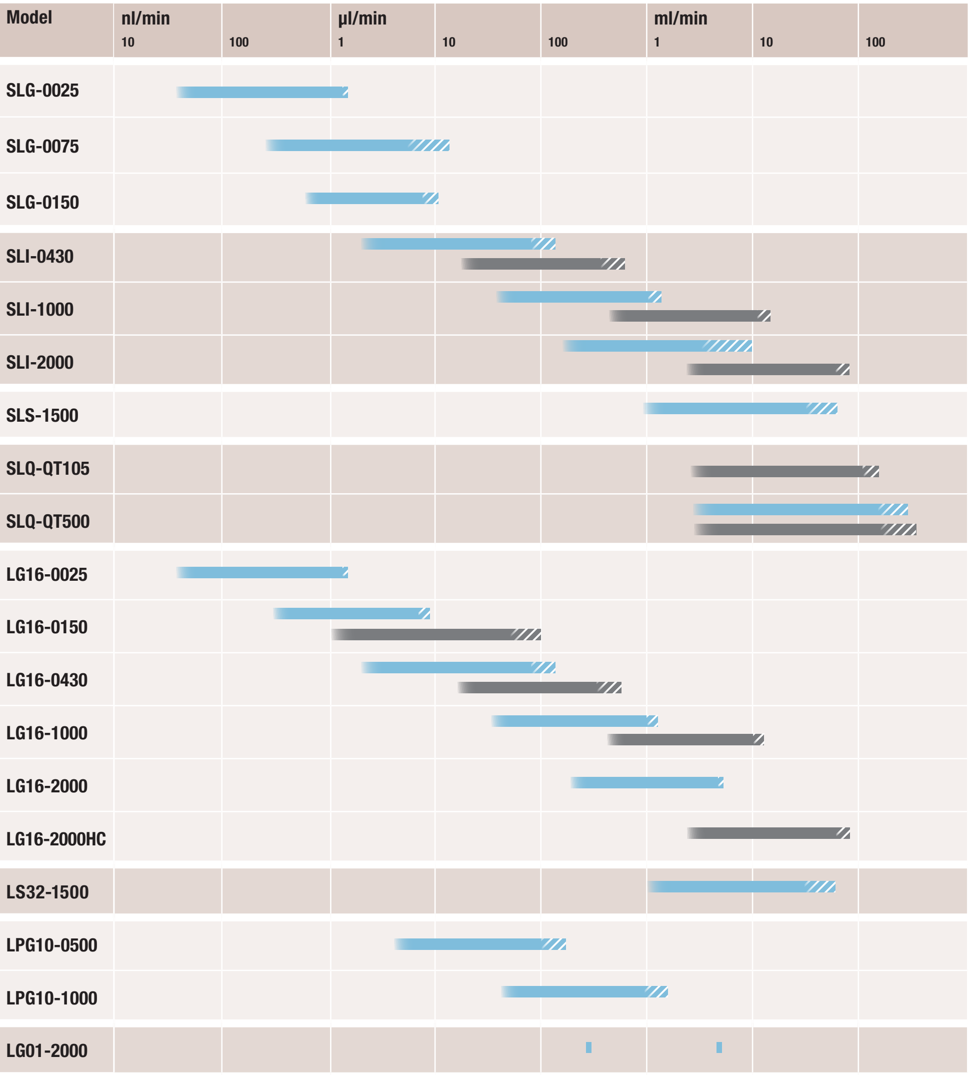

Calibrated flow rates for ■ water (H_2O) ■ hydrocarbons (HC)

Semiconductors

Analytical Instruments

Medical

Diagnostics

Automation

A Single-Use Liquid Flow Sensor – the LD20 Series

Nowadays, keywords and trends such as point-of-care, patient compliance, complex drug administration and wearable designs demand the development of smart medical devices. The single-use LD20 liquid flow sensor measures liquid flow rates of up to 1300 ml/h safely and reliably at the point of interest, enabling a more direct and effective patient treatment. The LD20 series combines Sensirion's proven liquid flow sensing technology with a single-use cost-effective design for high-volume applications in the biomedical, life sciences and food industry sectors.

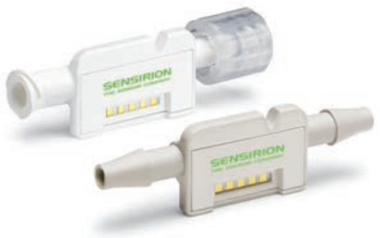

Detects failure modes during drug delivery (occlusion, air-in-line, infiltration...)

Liquid Flow Meter Kits

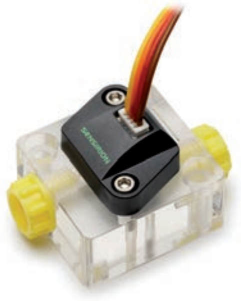

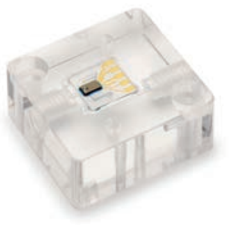

LPG10 Evaluation Kit

In order to conduct initial measurements quickly and easily, all our SLI, SLS, SLG, and SLQ liquid flow meters can be ordered as part of a Liquid Flow Meter Kit. In addition to the liquid flow meter of your choice, the kit also contains USB and analog interface cables for electrical connection, viewer and logging software, and a set of fluidic connectors. For liquid flow sensors in the LPG10 and LD20 series, we offer specific evaluation kits that include a pre-assembled manifold body for the LPG10 series or a base station for electrical connection of the contact pads for the LD20 series.

For more information please visit: www.sensirion.com/liquidflowmeterkit

Customized Solutions

Innovative ideas sometimes demand new solutions and Sensirion's sensor experts are always keen on finding ways to make interesting applications happen through our technology. Customized solutions can be designed for special requirements, such as flow rates, limited space constraints, resistance to aggressive chemicals, and dedicated low-price and even disposable sensor designs for high-volume applications.

Our cutting-edge sensor technology and unique expertise in liquid flow sensing has led to many successful customer projects. The essential goal is to understand the requirements of our customers and to implement the key benefits of our technology skillfully to their advantage: modification of the packaging, the wetted materials, the fluidic or electrical interface, improvement of the dead volume or pressure resistance of the sensor.

LOW FLOW RATES

Years of experience and expertise combined with the high sensitivity of our sensors enables flow measurement at extremely low ranges, from nanoliters to milliliters per minute.

HIGH SPEED

The MEMS sensor integrated on a CMOS chip permits ultra-fast response times – as fast as 20 ms – due to its small thermal mass.

FLEXIBILITY AND COST EFFICIENCY

With our advanced technology, we have the flexibility to address customer requirements to ensure a customized sensor solution that is both high-performance and cost-efficient.

1-900047-02 / 170608-LF-E

Sensing. Anytime. Anywhere.

SWITZERLAND
Sensirion AG
Laubisruetistrasse 50
8712 Staefa
Switzerland
Phone +41 44 306 40 00
Fax +41 44 306 40 30
info@sensirion.com
www.sensirion.com

UNITED STATES
Sensirion Inc.
11 East Adams, Suite 220
Chicago, IL 60603
United States
Phone +1 312 690 5858
info-us@sensirion.com
www.sensirion.com

CHINA
Sensirion China Co. Ltd.
Room 1006, Tower 1
Excellence Meilin Center Plaza (Excellence City)
ZhongKang Road Shangmeilin
Futian District, Shenzhen 518049
P.R. China
Phone +86 755 8252 1501
Fax +86 755 8252 1580
info-cn@sensirion.com
www.sensirion.com.cn

JAPAN
Sensirion Japan Co. Ltd.
Takanawa Kaneo Bldg. 4F
3-25-22, Takanawa Minato-ku, Tokyo
108-0074 Japan
Phone +81 3 3444 4940
Fax +81 3 3444 4939
info-jp@sensirion.com
www.sensirion.co.jp

KOREA
Sensirion Korea Co. Ltd.
14056, #1809-#1813 Gumkang Penterium A,
282, Hagui-Ro, Dongan-Gu
Anyang-Si, Gyeonggi-Do
South Korea
Phone +82 31 337 7700~3
Fax +82 31 337 7704
info-kr@sensirion.com
www.sensirion.co.kr

TAIWAN
Sensirion Taiwan Co. Ltd.
Rm. 2, 15F, No. 223, Fuxing 2nd Rd
Zhubei City
Hsinchu County, 30271
Taiwan, R.O.C.
Phone +886 3 5506701
Fax +886 3 5506703
info@sensirion.com
www.sensirion.com

SENSIRION
THE SENSOR COMPANY
