# Supplementary material for: Design of a Mechatronics Model of Urinary Bladder and Realization and Evaluation of Its Prototype
Source: Appl Bionics Biomech. 2019 Dec 14;2019:9431781. doi: 10.1155/2019/9431781 (PMC6948342; doi:10.1155/2019/9431781)
Supplement: Supplementary Materials — Among these 3 files, one is the product brochure, one is datasheet of the sensor, and the other is datasheet of the sensor cable. The datasheet of the sensor cable provides us the introduction of the sensor modes (in the manuscript, we use “Volume Counter” mode) and the connection diagram. The other two files are mainly about the introductions of the sensor such as specification charts and patented technology. [file 9431781.f1.zip › Sensirion_Liquid_Flow_Meters_SCC1-Analog_Sensor_Cable_V4.0.pdf]

Datasheet

Analog Sensor Cable

SCC1-Analog

- Configurable analog voltage output of flow rate
- Switch output with configurable threshold
- Volume integration by counting pulses
- Applicable to digital SLQ, SLI, SLS and SLG liquid flow meters

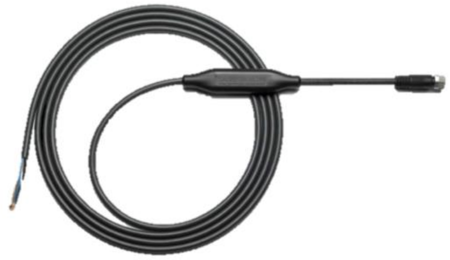

Product Summary

The Analog Sensor Cable SCC1-Analog allows simple and quick readout of Sensirion's liquid flow meters by converting the digital sensor reading to an analog voltage output configurable to any range within 0-10.5V. Additionally, a digital (high/low) output with two modes of operation is available (Flow Switch / Volume Counter).

In *Flow Switch* mode, the output is high or low depending on the momentary flow rate and two configurable threshold values.

In *Volume Counter* mode, a voltage pulse is generated every time a defined volume has flown through the flow meter.

Connecting the SCC1-Analog

One side of the SCC1-Analog is connected to the 4-Pin M8 connector of Sensirion's liquid flow meters. The other side has four wires: Two for power supply and one for each output (analog and digital output). The outputs should be measured as indicated in the block diagram of Fig. 7.

The interface electronics are molded into the cable. The cable is available in two length variants with a total length of 2 meters and 10 meters, respectively.

Wire Assignment

Wire	Function	Symbol
Blue	Supply voltage	V_{supp}
White	Ground	GND
Brown	Analog output	AOut
Black	Digital output	DOut

WARNING!
Incorrect connection may lead to permanent damage of the cable. Check the wire assignment carefully.

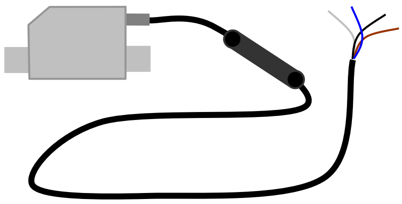

Fig. 1: SCC1-Analog connected to flow meter.

Analog Output (AOut)

The analog output provides a voltage which corresponds to the measured flow rate. The flow rate is linearly mapped to the voltage across a user-specified range, see Fig. 2, and can be calculated by the following formula:

$$flowrate = V_{AOut} \cdot \frac{flow_{at\mathrm{Vmax}} - flow_{at\ 0V}}{\mathrm{Vmax}} + flow_{at\ 0V}$$

The parameters Vmax, $flow_{atVmax}$ and $flow_{at0V}$ can be configured by the user (see section *Configuration*). They can be freely chosen in the range of the sensor's specifications (see appendix).
By default, SCC1-Analog is set with 5 V output at no-flow, 0 V for negative max flow, and 10 V for positive max flow. Refer to appendix for max flow

values by sensor model. The default calibration field accessed by SCC1-Analog can also be configured.
The sensor cable output voltage is capped at the Voltage output limit *Vlimit*. By default Vlimit is set to 10.5 V. See Fig. 2 below for an illustration of the different parameters.

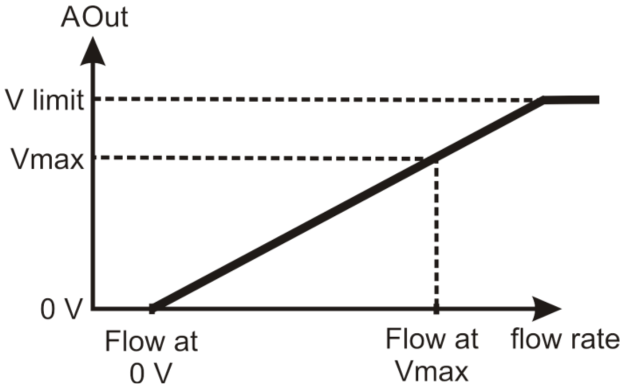

Fig. 2: Scaling of analog voltage output

Example:
The SLQ-QT500 has a maximum sensing range of +/-2500 µl/s. If the whole flow range is to be covered by the analog output, the two parameters $flow_{at0V}$ and $flow_{atVmax}$ are set to -2500 µl/s and +2500 µl/s, respectively, with Vmax being 10 V. A measured voltage of 8V at the analog output is then converted to the actual flow rate as follows:

$$8V \cdot \frac{2500\frac{ul}{s} - \left(-2500\frac{ul}{s}\right)}{10V} + \left(-2500\frac{ul}{s}\right) = 1500\frac{ul}{s}$$

Legacy information: On cables with date codes 1449 and lower the values for Vmax and Vlimit cannot be configured. The default values of 10.0 V and 10.5 V, respectively, are fixed.

Digital Output (DOut)

The digital output has only two logic states, high and low. Since the digital output is realized as an open collector circuit, a pull-up resistor has to be connected between digital output and an external voltage V_{high}, which serves as the high-level voltage (see block diagram in Fig. 7). The value of this high-level voltage may be chosen independently or identical to the supply voltage in order to match the logic levels of the customer system.

The different modes of operation for the digital output are described below. By default, the digital output is disabled, i. e. it is always low.
Other modes of operation may be configured using the configuration software. (See section C*onfiguration*).

Flow Switch

In this mode, the digital output is low when the measured flow is outside a specified flow range, and high when inside. This flow range is specified by two threshold values. Additionally, a hysteresis may be specified for each threshold value (see Fig. 3).

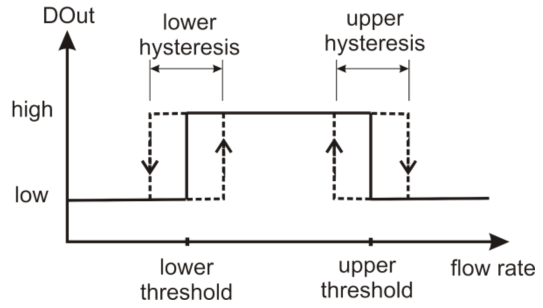

Fig. 3: Flow Switch thresholds

When the flow switch mode is first enabled in the configurator, a lower threshold at 25% of the maximum flow rate with a hysteresis of +-5% of the set point is suggested and the upper threshold is set above the maximum flow rate which is equivalent to disabling the upper threshold altogether.

Volume Counter

In this mode, the SCC1-Analog cable's internal electronics integrates the flow rate and every time a defined volume flows through the meter, a pulse is generated on the digital output. The total amount of liquid which flowed through the meter can be determined by simply counting the pulses and multiplying by the defined volume.

The liquid volume per output pulse and the duration of the output pulse can be configured.

The volume counter can be configured to ignore negative flow rates (flow in backward direction). If negative flow rates are not ignored, these are subtracted from the internally calculated volume. In this case, no output pulses are generated until the internal totalizer has again reached a positive value.

In order to reset the internal totalizer, the SCC1-Analog should be switched off for 1000 ms.

When the Volume Counter is first enabled in the configurator, the suggested setting generates 0.5 ms long output pulses with a frequency of approx. 250 Hz at the maximum flow rate of the attached sensor.

Output Filter *(for date codes 1450 and newer)*

Sensirion Liquid Flow sensors have a very fast response time and therefore react very quickly to changes of the flow rate. In some application a low-pass filtering of the sensor output may be desired e.g. to ignore short excursions of the flow rate above or below the switch thresholds. For this purpose, a moving average filter is available in the SCC1-Analog sensor cable (date codes 1450 and newer). The filter is realized as moving average with a configurable time constant (2 milliseconds to 1 minute). The moving average can be applied to the analog voltage output, to the digital output signal, to both outputs or to none of them. Figs 4, 5, and 6 show some examples of possible combinations and the resulting outputs on an oscillating flow rate with some interruptions of the flow. Such noisy flow rates are typical for certain pump types.

The filter time constant and signal selection (filtered/unfiltered) for each output may be configured using the configuration software. (See section C*onfiguration*).

Example 1:
Filtered signal for the analog voltage output, unfiltered signal for the digital (switch) output.

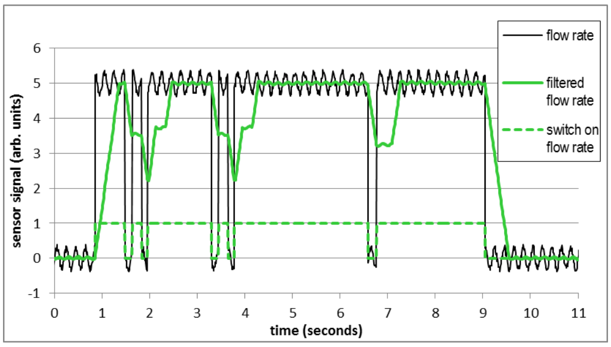

Fig. 4: Cable output with moving average filter on flow rate (green solid line) and switch output on unfiltered flow rate (green dashed line). The black line shows the underlying unfiltered flow rate.

Example 2:
Filtered signal for both, analog voltage output and digital (switch output). Note the difference in the switch behavior between Figs. 4 and 5: In Fig. 4 (unfiltered signal) the switch output follows the short dips in the flow rate immediately. In contrast, the switch output on the filtered signal (Fig. 5) is tolerant to such short excursions below the switch level.

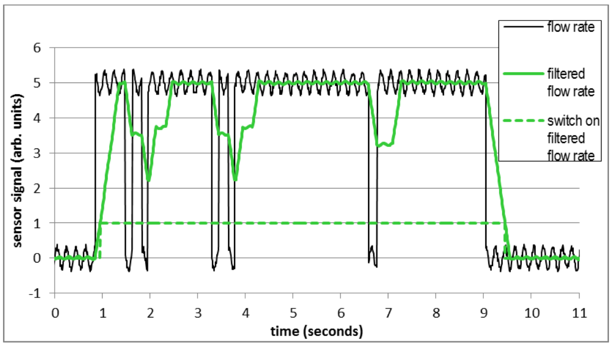

Fig. 5: Cable output with moving average filter on flow rate (green solid line) and switch output on filtered flow rate (green dashed line). The black line shows the underlying unfiltered flow rate.

Example 3:
Unfiltered signal for the analog voltage output, filtered signal for the digital (switch) output. The analog voltage output follows the flow rate immediately, including the high-frequency oscillations of the pump and the short drops in the flow rate. On the other hand, the switch output on the filtered signal is tolerant to these short excursions below the switch threshold.

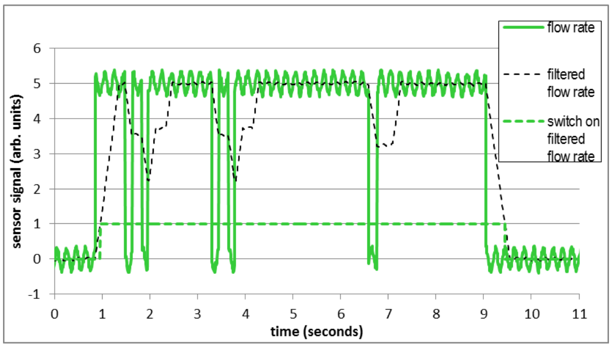

Fig. 6: Cable output with unfiltered flow rate (green solid line) and switch output on filtered flow rate (green dashed line). The black dashed line shows the underlying filtered flow rate.

Configuration

The output configuration settings for the SCC1-Analog are stored in the memory of the flow meter, not in the SCC1-Analog Sensor Cable. The configuration is written to the flow meter's memory using an SCC1-USB Sensor Cable and the configurator software which is available on the Sensirion webpage. Once the flow meter is configured, any SCC1-Analog can be connected to the flow meter. The internal electronics in the cable will then read the settings from the flow meter's memory and start operation on power-up.

The necessary steps are summarized below:

1) Connect the flow meter to a PC using the SCC1-USB Sensor Cable.
2) Open the Analog Sensor Cable Configurator software. Write your settings.
3) Disconnect the flow meter from the SCC1-USB Sensor Cable and reconnect with the SCC1-Analog. The SCC1-Analog will now read the output configuration from the flow meter and continuously update the output as soon as it is powered up.

Operating Conditions

Parameter	Min.	Typ.	Max.	Unit
Operating Temperature (*)	-25	25	85	°C
Storage Temperature	-40	25	105	°C

* Check also flow meter specifications for operating temperature of the flow meter.

Bending and Forces on Cable

Excessive, repetitive bending of the cable at the connections to the molded bulge may lead to cable breakage. Fasten the cable/bulge properly.

Electrical Characteristics

Parameter	Min.	Typ.	Max.	Unit
Supply Voltage	12	24	36	V
Power Consumption		104		mW
Voltage Analog Output	0		10.5	V
Current load Analog Output			10	mA
Accuracy Analog Output at 0 V*		5	20	mV
Accuracy Analog Output at 10 V*		5	40	mV
Current load Digital Output			20	mA
Pull-up Resistor Digital Output	2	10		kOhm
Low-level Digital Output		80	150	mV
High-level Digital Output **			24	V

* This is the accuracy of the digital-to-analog conversion in the SCC1-Analog. See the flow meter's datasheet for the measurement accuracy of the flow meter.
** Indicated as V_{high} in the block diagram.

Note on Resolution of the Analog Output

The internal DAC has 12-bit resolution, resulting in a theoretical resolution of the analog output of 2.6mV. In order to achieve this resolution on the output, a stabilized power supply should be used.

Note on Short Circuit Strength

The cable is in general not short circuit proof and does not have inverse-polarity protection. Incorrect connection of the cable may therefore cause damage to the cable.

The current at the digital output is limited by a thermal fuse resistor.

Note on Electromagnetic Interference

Strong electrical interference on the short distance between the flow meter and the cable electronics may disturb the digital communication between the cable and the sensor and should be avoided

Ordering Information

Product code	Product name	Article number
SCC1-Analog 2m	SCC1-Analog Sensor Cable, 2m length	1-101072-01
SCC1-Analog 10m	SCC1-Analog Sensor Cable, 10m length	1-101219-01

Connection Diagram

The voltages of the analog and digital outputs (V_{AOut} and V_{DOut}) should be measured with respect to GND, as indicated in the connection diagram of Fig. 7. In order to use the digital output, the DOut wire should be connected to the high voltage V_{high} by an external pull-up resistor $R_{pull-up}$ (typically 10 kOhm).

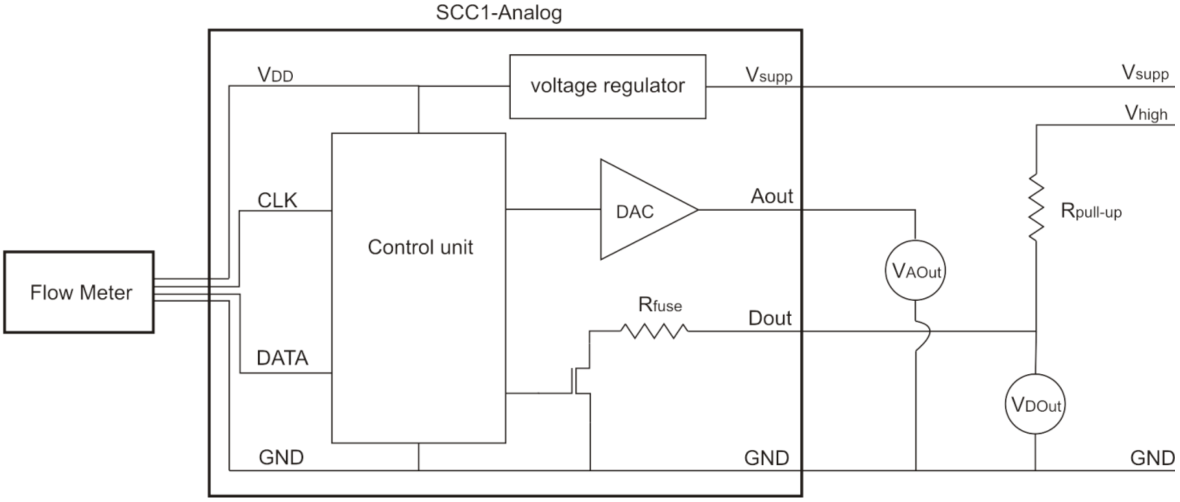

Fig 7: Internal block diagram of the SCC1-Analog Sensor Cable and measurement setup

Appendix: Max. Outputs for Sensor Types

The following tables list the minimum and maximum outputs of selected flow meter types as of April 2016. Values are subject to change. Please consult the latest version of your flow meter's datasheet on www.sensirion.com. Note that some flow meters have several different calibration fields (CF) available. The SCC1-Analog works with the default calibration field. The default can be changed with the USB/RS485 Sensor Viewer, version 2.30 and higher.

SLI-0430 *(Serial numbers 1440-00000 and higher)*

Medium	CF	min. output ($flow_{at0V}$)	max. output ($flow_{atVmax}$)
H_2O	0	-120 µl/min	120 µl/min
IPA	1	-600 µl/min	600 µl/min

SLI-1000

Medium	CF	min. output ($flow_{at0V}$)	max. output ($flow_{atVmax}$)
H_2O	0	-1100 µl/min	1100 µl/min
IPA	1	-11000 µl/min	11000 µl/min

SLI-2000

Medium	CF	min. output ($flow_{at0V}$)	max. output ($flow_{atVmax}$)
H_2O	0	-5500 µl/min	5500 µl/min
IPA	1	-90 ml/min	90 ml/min

SLG-0025

Medium	CF	min. output ($flow_{at0V}$)	max. output ($flow_{atVmax}$)
H_2O	0	-1700 nl/min	1700 nl/min

SLG-0075

Medium	CF	min. output ($flow_{at0V}$)	max. output ($flow_{atVmax}$)
H_2O	0	-5500 nl/min	5500 nl/min
H_2O (extended)	1	1800 nl/min	22000 nl/min

SLG-0150

Medium	CF	min. output ($flow_{at0V}$)	max. output ($flow_{atVmax}$)
H_2O	0	-10500 nl/min	10500 nl/min

SLQ-QT105

Medium	CF	min. output ($flow_{at0V}$)	max. output ($flow_{atVmax}$)
IPA	0	-2400 µl/s	2400 µl/s

SLQ-QT500

Medium	CF	min. output ($flow_{at0V}$)	max. output ($flow_{atVmax}$)
IPA	0	-2500 µl/s	2500 µl/s
H_2O	2	-2500 µl/s	2500 µl/s

SLS-1500

Medium	CF	min. output ($flow_{at0V}$)	max. output ($flow_{atVmax}$)
H_2O	0	-65 ml/min	65 ml/min

Important Notices

Warning, personal injury

Do not use this product as safety or emergency stop devices or in any other application where failure of the product could result in personal injury. Do not use this product for applications other than its intended and authorized use. Before installing, handling, using or servicing this product, please consult the data sheet and application notes. Failure to comply with these instructions could result in death or serious injury.

If the Buyer shall purchase or use SENSIRION products for any unintended or unauthorized application, Buyer shall defend, indemnify and hold harmless SENSIRION and its officers, employees, subsidiaries, affiliates and distributors against all claims, costs, damages and expenses, and reasonable attorney fees arising out of, directly or indirectly, any claim of personal injury or death associated with such unintended or unauthorized use, even if SENSIRION shall be allegedly negligent with respect to the design or the manufacture of the product.

ESD Precautions

The inherent design of this component causes it to be sensitive to electrostatic discharge (ESD). To prevent ESD-induced damage and/or degradation, take customary and statutory ESD precautions when handling this product.

Warranty

SENSIRION warrants solely to the original purchaser of this product for a period of 12 months (one year) from the date of delivery that this product shall be of the quality, material and workmanship defined in SENSIRION's published specifications of the product. Within such period, if proven to be defective, SENSIRION shall repair and/or replace this product, in SENSIRION's discretion, free of charge to the Buyer, provided that:

- notice in writing describing the defects shall be given to SENSIRION within fourteen (14) days after their appearance;
- such defects shall be found, to SENSIRION's reasonable satisfaction, to have arisen from SENSIRION's faulty design, material, or workmanship;
- the defective product shall be returned to SENSIRION's factory at the Buyer's expense; and
- the warranty period for any repaired or replaced product shall be limited to the unexpired portion of the original period.

This warranty does not apply to any equipment which has not been installed and used within the specifications recommended by SENSIRION for the intended and proper use of the equipment. EXCEPT FOR THE WARRANTIES EXPRESSLY SET FORTH HEREIN, SENSIRION MAKES NO WARRANTIES, EITHER EXPRESS OR IMPLIED, WITH RESPECT TO THE PRODUCT. ANY AND ALL WARRANTIES, INCLUDING WITHOUT LIMITATION, WARRANTIES OF MERCHANTABILITY OR FITNESS FOR A PARTICULAR PURPOSE, ARE EXPRESSLY EXCLUDED AND DECLINED.

SENSIRION is only liable for defects of this product arising under the conditions of operation provided for in the data sheet and proper use of the goods. SENSIRION explicitly disclaims all warranties, express or implied, for any period during which the goods are operated or stored not in accordance with the technical specifications.

SENSIRION does not assume any liability arising out of any application or use of any product or circuit and specifically disclaims any and all liability, including without limitation consequential or incidental damages. All operating parameters, including without limitation recommended parameters, must be validated for each customer's applications by customer's technical experts. Recommended parameters can and do vary in different applications.

SENSIRION reserves the right, without further notice, (i) to change the product specifications and/or the information in this document and (ii) to improve reliability, functions and design of this product.

Copyright© 2001-2016, SENSIRION.
CMOSens® is a trademark of Sensirion
All rights reserved.

RoHS and WEEE Statement

The Analog sensor cable complies with requirements of the following directives:

- EU Directive 2002/96/EC on waste electrical and electronic equipment (**WEEE**), OJ13.02.2003; esp. its Article 6 (1) with Annex II.
- EU Directive 2002/95/EC on the restriction of the use of certain hazardous substances in electrical and electronic equipment (**RoHS**), OJ 13.02.2003; esp. its Article 4.

Headquarter and Sales Offices

SENSIRION AG
Laubisruetistr. 50
CH-8712 Staefa ZH
Switzerland

phone: +41 44 306 40 00
fax: +41 44 306 40 30
info@sensirion.com
www.sensirion.com

Sensirion Inc., USA
phone: +1 805 409 4900
info_us@sensirion.com
www.sensirion.com

Sensirion Japan Co. Ltd.
phone: +81 3 3444 4940
info-jp@sensirion.com
www.sensirion.co.jp

Sensirion Korea Co. Ltd.
phone: +82 31 345 0031 3
info-kr@sensirion.com
www.sensirion.co.kr

Sensirion China Co. Ltd.
phone: +86 755 8252 1501
info-cn@sensirion.com
www.sensirion.com.cn

Sensirion Taiwan Co. Ltd.
info@sensirion.com
www.sensirion.com

To find your local representative, please visit www.sensirion.com/contact
