# Supplementary material for: Design of a Mechatronics Model of Urinary Bladder and Realization and Evaluation of Its Prototype
Source: Appl Bionics Biomech. 2019 Dec 14;2019:9431781. doi: 10.1155/2019/9431781 (PMC6948342; doi:10.1155/2019/9431781)
Supplement: Supplementary Materials — Among these 3 files, one is the product brochure, one is datasheet of the sensor, and the other is datasheet of the sensor cable. The datasheet of the sensor cable provides us the introduction of the sensor modes (in the manuscript, we use “Volume Counter” mode) and the connection diagram. The other two files are mainly about the introductions of the sensor such as specification charts and patented technology. [file 9431781.f1.zip › Sensirion_Liquid_Flow_Meters_SLI_Datasheet_V11.pdf]

# SLI Liquid Flow Meter Series

## Media Isolated Microfluidic Flow Meter

- Liquid flow rates up to 10 ml/min
- Non-invasive measurement
- Different interface options
- 40 ms flow detection response time

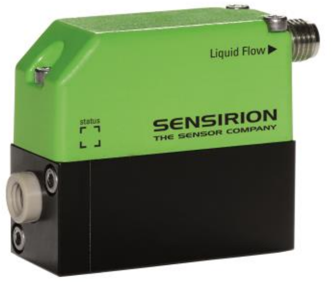


### Product Summary

The SLI Liquid Flow Meter enables fast, non-invasive measurements of very low liquid flow in the µl/min- to ml/min-range. Excellent chemical resistance is ensured: The flow path of the SLI Liquid Flow Meter is formed by a simple, straight glass capillary. The fourth generation MEMS sensors combine a thermal high precision sensor element with amplification circuits and digital intelligence for linearization and temperature compensation on one single microchip – the product's core element.

### Interface Options

**Digital**
- $I^2C$-Bus
- RS485-Bus
- USB Cable

**Analog**
- Voltage Output (0-10 V)
- Additional operation modes

For more information on communication, please refer to page 2 of this document.

## 1 Sensing Performance

Table 1: Model specific performance of SLI (all data for medium **$H_2O$**, 23°C)

| Parameter | SLI-0430 | SLI-1000 | SLI-2000 | Unit |
|---|---|---|---|---|
| $H_2O$ Full scale flow rate | 80 | 1000 | 5000 | µl/min |
| $H_2O$ Sensor output limit[a] | 120 | 1100 | 5500[b] | µl/min |
| Accuracy below full scale<br>(whichever error is larger) | 5.0<br>0.15 | 5.0<br>0.2 | 5.0<br>0.2 | % of m.v.[c]<br>% of full scale |
| Repeatability below full scale<br>(whichever error is larger) | 0.5<br>0.01 | 0.5<br>0.02 | 0.5<br>0.02 | % of m.v.<br>% of full scale |
| Temperature coefficient<br>(additional error / °C; whichever is larger) | 0.13<br>0.003 | 0.1<br>0.004 | 0.1<br>0.004 | % m.v. / °C<br>% full scale / °C |
| Mounting orientation sensitivity[d] | <0.4 | 1.0 | 1.5 | % of full scale |
| Flow detection response time $\tau_{63}$ | 40 | | | ms |
| Response time on power-up | 120 | | | ms |
| Operating temperature | +10…+50 | | | °C |
| Ambient storage temperature[e] | -10…+60 | | | °C |
| Maximum recommended operating pressure | 50 | 15 | 15 | bar |
| Burst pressure | 150 | 30 | 30 | bar |

[a] Flow rate at which the sensor output saturates. See section 2 for performance between full scale and saturation point
[b] Extended range up to 10500 µl/min, see section 2 for performance specifications
[c] Measured value
[d] Maximum additional offset when mounted vertically
[e] Non-condensing, flow path empty

Table 2: Model specific performance of SLI (all data for medium **IPA**, 23°C)

| Parameter | SLI-0430 | SLI-1000 | SLI-2000 | Unit |
|---|---|---|---|---|
| IPA full scale flow rate | 500 | 10’000 | | µl/min |
| | | | 80 | ml/min |
| Sensor output limit[a] | 600 | 11’000 | | µl/min |
| | | | 90 | ml/min |
| Accuracy below full scale<br>(whichever error is larger) | 20<br>1 | 20<br>1 | 10<br>0.5 | % of m.v.[b]<br>% of full scale |
| Repeatability below full scale<br>(whichever error is larger) | 1<br>0.05 | 1<br>0.05 | 1.5<br>0.03 | % of m.v.<br>% of full scale |
| Temperature coefficient<br>(additional error / °C; whichever is larger) | 0.5<br>0.025 | 0.4<br>0.02 | 0.35<br>0.02 | % m.v. / °C<br>% full scale / °C |

[a]Flow rate at which the sensor output saturates
[b]Measured value

## 1.1 Calibration Field Information

The SLI Liquid Flow Meters hold calibrations for two liquids, one for water ($H_2O$) and one for isopropyl alcohol (IPA). Each calibration is stored on a separate calibration field (CF):

- Calibration field 0: $H_2O$ (factory default)
- Calibration field 1: IPA
- Calibration field 2 (SLI-2000 only, starting from SN 1627-00000): $H_2O$ extended range

The default calibration field (i.e. the active calibration field at power up) can be permanently changed via I²C or RS485 commands. Alternatively, the default calibration field can be changed using the USB-RS485 Sensor Viewer which is part of the Liquid Flow Meter Kit and also available in the download center on the Sensirion liquid flow webpage. www.sensirion.com/liquidflow-download

# 2 Specifications Charts

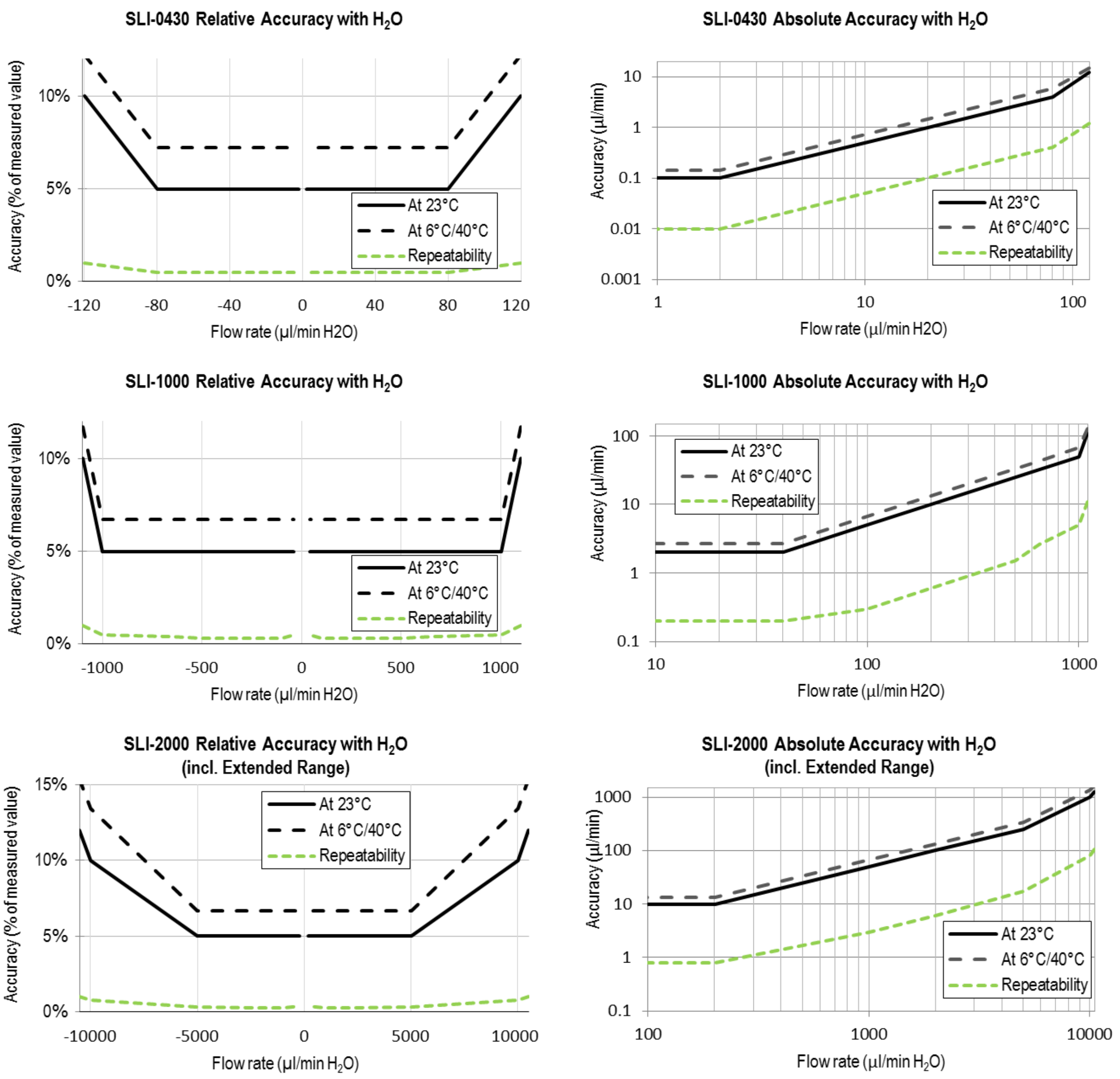


Figure 1: Flow meter accuracy and repeatability across the flow range. Relative error in % of measured value (left column) and absolute error in µl/min (right column) for $H_2O$

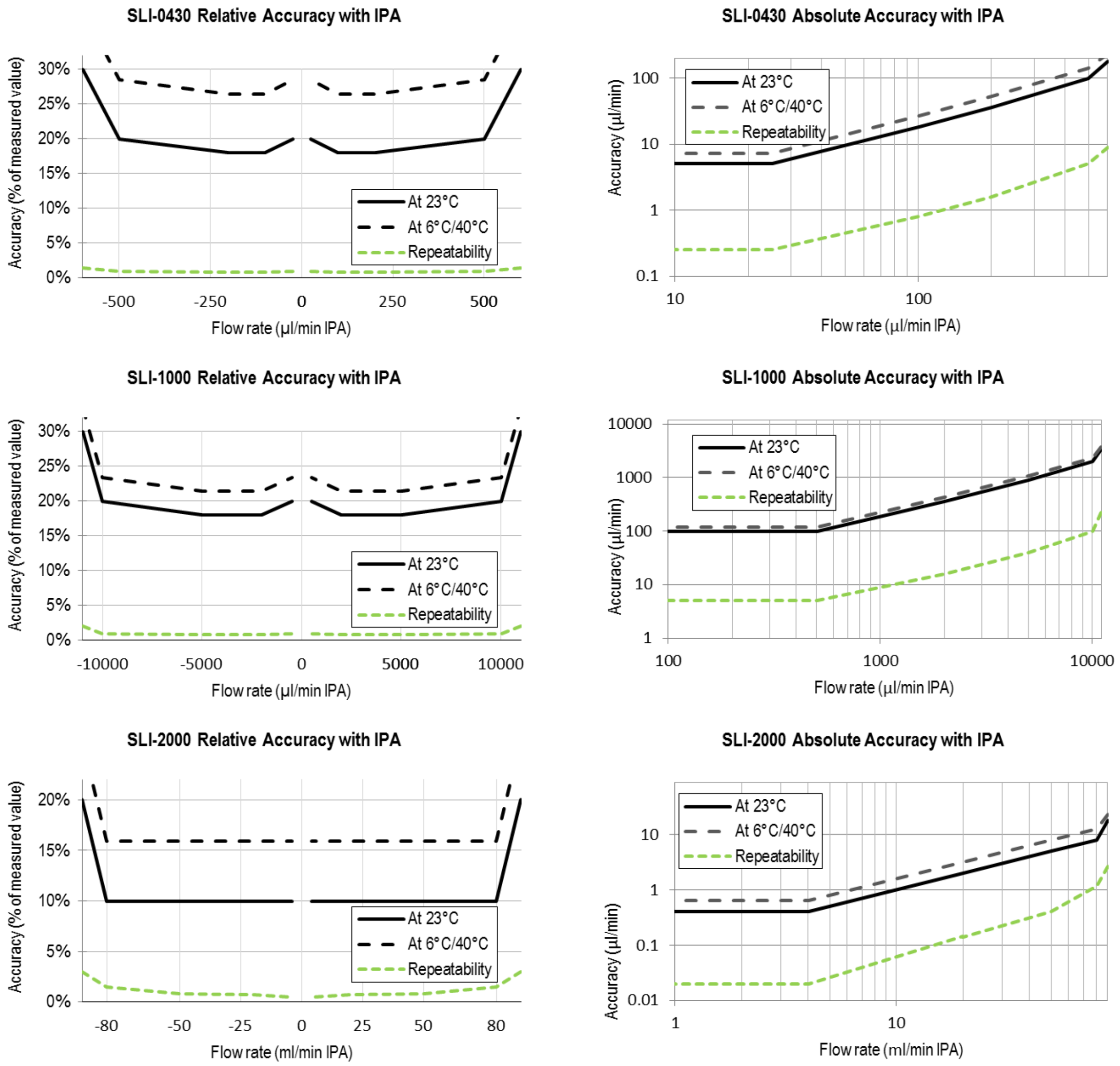


Figure 2: Flow meter accuracy and repeatability across the flow range. Relative error in % of measured value (left column) and absolute error in µl/min or ml/min in case of the SLI-2000 (right column) for IPA

# 3 Communication with the Sensor

The SLI flow meter shows bidirectional, linear transfer characteristics. The product comes fully calibrated for water and IPA.

| | |
|---|---|
| Digital Sampling Time, 16 bit | 74 ms |
| Digital Sampling Time, 9 bit | 1 ms |

## 3.1 Electrical Specifications

Table 3: DC Characteristics

| Parameter | Conditions | Min. | Typ. | Max. | Units |
|---|---|---|---|---|---|
| Power Supply DC, VDD | Sensor only | 4 | 5 | 6 | V |
| | RS485 cable | 4 | 5 | 6 | V |
| | Analog cable | 12 | 24 | 36 | V |
| Operating Current | VDD = 4-6 V, no load | 5 | 5.5 | 6 | mA |
| | RS485 cable | | 20 | 70 | mA |
| | Analog cable | | 4.3 | | mA |

## 3.2 Electrical Connector and Pinout

The flow meter is equipped with a male connector type M8, 4-pin, threaded lock according to IEC 61076-2-101 (Ed. 1)/ IEC 60947-5-2, and is compatible with Sensirion's SCC1 interface cables.

Table 3: Electrical pinout

| Pin | |
|---|---|
| 1 | SDA (data) |
| 2 | GND |
| 3 | VDD |
| 4 | SCL (clock) |

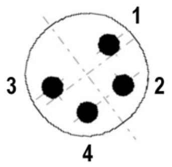


## 3.3 Communication via USB cable

The Sensirion USB Sensor Cable provides an easy to use USB Interface for laboratory and desktop use.

For further information please see the SCC1-USB Sensor Cable datasheet, available on www.sensirion.com/liquidflow-download.

## 3.4 Digital Communication via RS485-Bus

The SCC1-RS485 Sensor Cable for flow sensors allows the communication via RS485 interface for use in a demanding industrial automation environment. In addition to the standard commands available in the I²C interface of the sensor, the incorporated microcontroller of the cable provides more complex logic such as a dispense volume totalizer, automatic dispense detection, automatic heater control and data buffer for asynchronous read-out.

For further information please see the SCC1-RS485 Sensor Cable datasheet, available on www.sensirion.com/liquidflow-download.

## 3.5 Analog Communication

The SCC1-ANALOG Sensor Cable allows simple and quick readout of Sensirion's liquid flow meters by converting the digital sensor reading to a 0...10.5 V analog voltage output. Additionally, a digital (high/low) output with two different modes of operation is available (Flow Switch / Volume Counter)

For further information please see the SCC1-USB Sensor Cable datasheet, available on www.sensirion.com/liquidflow-download.

## 3.6 Digital Communication via I²C-Bus

Digital communication between a master and the SLI sensor runs via the standard I²C-interface. The physical interface consists of two bus lines, a data line (SDA) and a clock line (SCL) which need to be connected via pull-up resistors to the bus voltage of the system.

These lines can be used on 3.3V or 5.0V level with a clock frequency of 100 kHz. For the detailed specifications of this I²C communication, please refer to specific I²C Application Notes from Sensirion.

# 4 Fluidic Connection

Table 5: Fluidic Specifications and Pressure Rating

| Parameter | SLI-0430 | SLI-1000 | SLI-2000 |
|---|---|---|---|
| Wetted Materials: | | | |
| • Internal sensor tube material | Quartz Glass (Fused Silica) | Borosilicate Glass 3.3 | |
| • Fitting material | PEEK | | |
| • Sealing material | None | FEP | |
| Fluid connector ports (Fittings) | ¼-28 flat-bottom for 1/16” or 1/8” OD plastic tubing[a] | | |
| Pressure drop (at full scale flow rate, $H_2O$, 23°C) | 1 mbar | <1 mbar | <1 mbar |
| Pressure drop (at full scale flow rate, IPA, 23°C) | 7 mbar | 5 mbar | 2 mbar |
| Total internal volume | 5 µl | 25 µl | 80 µl |

[a] 1/8” OD tubing with 2 mm minimum ID is recommended for the SLI-2000.

For more information on the fluidic connection please find: “Application Note Sensor Ports and Tubing Connections” in the Download Center on our webpage (www.sensirion.com/liquidflow-download).

# 5 Mechanical Specifications

Table 6: Mechanical Specifications

| Parameter | SLI-0430 | SLI-1000 | SLI-2000 |
|---|---|---|---|
| Largest dimensions | 58 x 42.5 x 20 mm | | |
| Total mass | 53 g | | |
| Inner diameter d | 430 µm | 1.0 mm | 1.8 mm |
| Protection class | IP 65 | | |

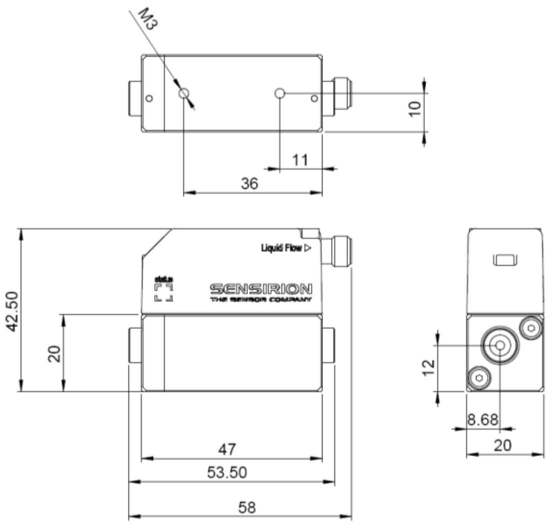


SLI-0430

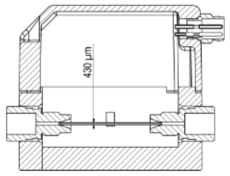


SLI-1000 / SLI-2000

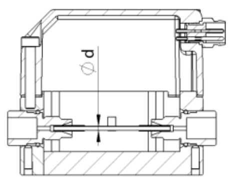


**All dimensions in mm**

# 6 Ordering Information

For OEM applications, the liquid flow meter can be purchased in larger quantities without any additional parts. For optimum performance, Sensirion recommends using the SLI liquid flow meters in combination with the SCC1 interface cables.

For laboratory use and technology evaluation, the Liquid Flow Meter Kit SLI-XXXX can be ordered.

This laboratory-package contains:

- Liquid Flow Meter SLI-XXXX
- Fitting Material for 1/8'' tubing
- PC Software (Viewer & Data Export Tool)
- SCC1-USB Sensor Cable with USB connector for plug-and-play connection to a PC
- SCC1-Analog Sensor Cable with 0-10 V voltage output.

Table 9: Ordering information

| Product | Article Number |
|---|---|
| SLI-0430 Liquid Flow Meter | 1-100836-02 |
| SLI-1000 Liquid Flow Meter | 1-100835-01 |
| SLI-2000 Liquid Flow Meter | 1-100895-01 |
| Flow Meter Kit SLI-0430 | 1-100893-01 |
| Flow Meter Kit SLI-1000 | 1-100879-01 |
| Flow Meter Kit SLI-2000 | 1-100894-01 |

Interface Cables:

| | |
|---|---|
| SCC1-RS485 Sensor Cable Pigtail 2m | 1-100804-01 |
| SCC1-RS485 Sensor Cable Pigtail 5m | 1-101122-01 |
| SCC1-ANALOG Sensor Cable Pigtail 2m | 1-101072-01 |
| SCC1-ANALOG Sensor Cable Pigtail 10m | 1-101219-01 |
| SCC1-USB Sensor Cable 2m | 1-101007-01 |

# Important Notices

**Warning, personal injury**

**Do not use this product as safety or emergency stop devices or in any other application where failure of the product could result in personal injury (including death). Do not use this product for applications other than its intended and authorized use. Before installing, handling, using or servicing this product, please consult the datasheet and application notes. Failure to comply with these instructions could result in death or serious injury.**

If the Buyer shall purchase or use SENSIRION products for any unintended or unauthorized application, Buyer shall defend, indemnify and hold harmless SENSIRION and its officers, employees, subsidiaries, affiliates and distributors against all claims, costs, damages and expenses, and reasonable attorney fees arising out of, directly or indirectly, any claim of personal injury or death associated with such unintended or unauthorized use, even if SENSIRION shall be allegedly negligent with respect to the design or the manufacture of the product.

**ESD Precautions**

The inherent design of this component causes it to be sensitive to electrostatic discharge (ESD). To prevent ESD-induced damage and/or degradation, take customary and statutory ESD precautions when handling this product.

**Warranty**

SENSIRION warrants solely to the original purchaser of this product for a period of 12 months (one year) from the date of delivery that this product shall be of the quality, material and workmanship defined in SENSIRION's published specifications of the product. Within such period, if proven to be defective, SENSIRION shall repair and/or replace this product, in SENSIRION's discretion, free of charge to the Buyer, provided that:

- notice in writing describing the defects shall be given to SENSIRION within fourteen (14) days after their appearance;
- such defects shall be found, to SENSIRION's reasonable satisfaction, to have arisen from SENSIRION's faulty design, material, or workmanship;
- the defective product shall be returned to SENSIRION's factory at the Buyer's expense; and
- the warranty period for any repaired or replaced product shall be limited to the unexpired portion of the original period.

This warranty does not apply to any equipment which has not been installed and used within the specifications recommended by SENSIRION for the intended and proper use of the equipment. EXCEPT FOR THE WARRANTIES EXPRESSLY SET FORTH HEREIN, SENSIRION MAKES NO WARRANTIES, EITHER EXPRESS OR IMPLIED, WITH RESPECT TO THE PRODUCT. ANY AND ALL WARRANTIES, INCLUDING WITHOUT LIMITATION, WARRANTIES OF MERCHANTABILITY OR FITNESS FOR A PARTICULAR PURPOSE, ARE EXPRESSLY EXCLUDED AND DECLINED.

SENSIRION is only liable for defects of this product arising under the conditions of operation provided for in the datasheet and proper use of the goods. SENSIRION explicitly disclaims all warranties, express or implied, for any period during which the goods are operated or stored not in accordance with the technical specifications.

SENSIRION does not assume any liability arising out of any application or use of any product or circuit and specifically disclaims any and all liability, including without limitation consequential or incidental damages. All operating parameters, including without limitation recommended parameters, must be validated for each customer's applications by customer's technical experts. Recommended parameters can and do vary in different applications.

SENSIRION reserves the right, without further notice, (i) to change the product specifications and/or the information in this document and (ii) to improve reliability, functions and design of this product.

Copyright © 2001-2016, SENSIRION.

CMOSens® is a trademark of Sensirion

All rights reserved

**CE, RoHS, REACH and WEEE Statement**

The flow meters of the SLI series comply with requirements of the following directives and regulations:

- The device fully complies with norm EN 50081-2 (Emission Test Series), EN 50082-2 (Immunity Test Series) and ESD protection when used in combination with the SCC1-RS485 or SCC1-ANALOG Sensor Cables.
- EU Directive 1907/2006/EC concerning Registration, Evaluation, Authorization and Restriction of Chemicals (REACH)
- EU Directive 2002/96/EC on waste electrical and electronic equipment (WEEE), OJ13.02.2003; esp. its Article 6 (1) with Annex II.
- EU Directive 2002/65/EC on the restriction of certain hazardous substances in electric and electronic equipment (RoHS), OJ01.01.2011

# Headquarters and Subsidiaries

SENSIRION AG
Laubisruetistr. 50
CH-8712 Staefa ZH
Switzerland

phone: +41 44 306 40 00
fax: +41 44 306 40 30
info@sensirion.com
www.sensirion.com

Sensirion Taiwan Co. Ltd.
info@sensirion.com
www.sensirion.com

Sensirion Inc., USA
phone: +1 805 409 4900
info-us@sensirion.com
www.sensirion.com

Sensirion Japan Co. Ltd.
phone: +81 3 3444 4940
info-jp@sensirion.com
www.sensirion.co.jp

Sensirion Korea Co. Ltd.
phone: +82 31 337 7700-3
info-kr@sensirion.com
www.sensirion.co.kr

Sensirion China Co. Ltd.
phone: +86 755 8252 1501
info-cn@sensirion.com
www.sensirion.com.cn

To find your local representative, please visit www.sensirion.com/contact
